# Targeted allelic diversification of flowering genes via CRISPR-Cas enables the development of multiple early-flowering soybean lines

**DOI:** 10.1007/s00122-026-05400-9

**Published:** 2026-09-29

**Authors:** Manon Monfort, Norbert Brugière, Julie Calbry, Laurène Lacou, Frank Roeber, Fabien Nogué

**Affiliations:** 1 Université Paris-Saclay, INRAE, AgroParisTech, Institute Jean-Pierre Bourgin for Plant Sciences, 78000 Versailles, France; 2 Biotechnology, Corteva Agriscience™, 62nd Ave, Johnston, IA 50131 USA; 3 Corteva Agriscience, 1131, Chemin de L’Enseigure, 31840 Aussonne, France; 4 Corteva Agriscience France, 1601 Rte de Lalande, 40400 Carcarès-Sainte-Croix, France; 5 Corteva Agriscience Germany GmbH, Riedenburgerstr. 7, 81677 Munich, Germany

## Abstract

*****Key message***:**

**CRISPR-Cas multiplex editing generated a broad range of early-flowering soybean lines, advancing flowering by up to four weeks under controlled long-day conditions and by approximately one to two weeks in a field trial.**

**Abstract:**

Soybean is a promising crop for European agriculture, but its cultivation area remains smaller than its potential. A major limitation to broader adoption in Europe is the restricted number of elite lines adapted to local environmental conditions, particularly the photoperiod encountered at higher latitude. At these latitudes, soybean must flower early to complete its growth cycle before the onset of harsh autumn conditions. In this project, we employed a CRISPR-Cas multiplex knockout strategy, to generate allelic diversity in the gene network controlling flowering time, with the aim of developing earlier flowering soybeans lines better suited to European high latitudes. This approach successfully produced earlier flowering varieties, with flowering advances of up to four weeks compared to the controls. Starting from maturity group (MG) I and MG III lines, we developed, within two generations, gene-edited early flowering soybean lines with estimated maturity ranges between MG 000 and MG 0. Greenhouse observations were supported by a field trial, in an MG II environment, where edited lines flowered approximately one to two weeks earlier than their respective parental controls. These results demonstrate that multiplex editing of flowering-time genes can rapidly generate targeted allelic diversity and a broad spectrum of flowering phenotypes within elite soybean backgrounds. These results provide valuable breeding material and a genotype/phenotype resource for subsequent evaluation of flowering-time adaptation across environments.

**Supplementary Information:**

The online version contains supplementary material available at https://doi.org/10.1007/s00122-026-05400-9.

## Introduction

Soybean [*Glycine max* (L.) Merr.] is a major source of plant protein and oil worldwide. In recent years, its cultivation has gained a growing interest in Europe. However, as a short-day plant, soybean flowering and subsequent maturity are strongly influenced by photoperiod (Garner and Allard 1920), limiting its expansion to high-latitude environments characterized by long summer days (Kurasch et al. 2017).

During domestication, selection for flowering time enabled adaptation across a broad range of latitudes. However, this photoperiod sensitivity still restricts most cultivars to a narrow latitudinal range (~ 200 km) (Scott et al. 1986). To optimize yield, cultivars in lower latitudes benefit from delayed flowering, whereas those in higher latitudes must flower earlier to complete their growth cycle before the onset of unfavorable autumn conditions. To classify this adaptation, soybean cultivars are grouped into 14 maturity groups (MGs), ranging from MG 0000 in higher latitudes to MG X in lower latitudes, reflecting their flowering response to day length and, consequently, their geographic suitability (Hymowitz 2008).

These differences in adaptation among soybean cultivars are controlled by the genetic variation within flowering-time regulatory pathways. Several genetic factors regulating flowering time and maturity have been identified in soybean, including the eleven classical E maturity loci (E1–E11) (Buzzell 1971; Bernard 1971; Buzzell and Voldeng 1980; Mcblain and Bernard 1987; Bonato and Vello 1999; Cober and Voldeng 2001; Cober et al. 2010; Kong et al. 2014; Samanfar et al. 2017; Wang et al. 2019). In addition, several flowering-time genes with known molecular identities have been characterized, including *GmCOL1a* and *GmCOL1b* (Cao et al. 2015)*, J* (Ray et al. 1995), *Tof11 (GmPRR3a)* and *Tof12 (GmPRR3b)* (Li et al. 2020, 2019; Lu et al. 2020), *Tof16* (Dong et al. 2021) or *Tof5* (Dong et al. 2022). Beyond these, multiple quantitative trait loci (QTLs) associated with flowering time and maturity have also been reported*.* While natural allelic variation at some of these loci has been exploited in breeding, the overall allelic diversity available, particularly for flowering repressors, remains limited, constraining fine-tuning of flowering time across diverse environments.

In recent years, CRISPR-Cas9 genome editing tools have enabled tremendous advancements in both fundamental research and crop improvement. In soybean, CRISPR-based approaches have been successfully applied to modify flowering time, often by targeting one or a small number of key genes such as *E1* or its homologs (Zhu et al. 2019; Gao et al. 2024). However, flowering time is a polygenic trait, and single-gene knockouts frequently produce discrete phenotypic shifts rather than the continuous variation required for optimal adaptation across latitude gradients. Moreover, focusing exclusively on predefined major-effect genes risks overlooking beneficial allele combinations within the broader flowering regulatory network.

An alternative strategy is to generate targeted genetic diversity within an entire developmental pathway and subsequently identify favorable allele combinations through phenotypic screening. Targeted Induced Gene Variation (TIGV, Nogué et al. 2016) was proposed as a framework to achieve this goal by combining multiplex genome editing with unbiased phenotypic selection. By simultaneously editing multiple genes within a defined pathway, TIGV enables the rapid creation of a wide spectrum of allelic combinations, mimicking and extending natural variation while remaining confined to biologically relevant targets.

In this study, we employed a TIGV-inspired CRISPR-Cas9 multiplex strategy to engineer flowering-time diversity in soybean while preserving valuable agronomic traits. This approach successfully yielded early flowering lines, which were evaluated under both controlled greenhouse conditions and in the field. By extending our analysis to field conditions, this study underscores both the potential and the limitations of greenhouse-based evaluations of soybean flowering time, while also revealing the broader agronomic consequences of editing the flowering pathway, including effects on plant maturity, yield performance, and seed oil and protein content. This study tested whether multiplex CRISPR-Cas9 editing of multiple flowering-time repressors could generate useful phenotypic diversity within elite soybean backgrounds and whether selected early-flowering genotypes would retain early flowering under field conditions. We further investigated the relationship between the resulting allelic combinations and flowering-time phenotypes to identify gene combinations associated with precocious flowering.

## Materials and methods

### Plant material and growth conditions

Corteva Agriscience proprietary commercial soybean cultivars 91B42 from MG I and 93Y21 from MG III were used for this study. To identify the maturity group of gene-edited lines, commercial varieties with a range of MGs have been commercially acquired and used as comparators. Commercial varieties used in greenhouse experiments included, P21T45 (MG I, Corteva), P18A02 (MG I, Corteva), Ginevra (MG 0.5, Syngenta), Cinzia (MG 0, APSOVSEMENTI), ES Mentor (MG 00, Lidea), ES Comandor (MG 000, Lidea), Williams 82 (MG III, courtesy of Dr. Julia Buitink, INRAE Angers). Varieties used in the field trial all came from Corteva Agriscience and included commercial P02A78E (MG 0), P05Z60E (MG 0), P08A44E (MG 0), P002A42E (MG 00), P004Z87E (MG 00), P007A68E (MG 00), P0009A28E (MG 000), and two pre-commercial (PC1 and PC2) MG 000 lines.

### Guide RNA (gRNA) design

For the selection of the 20-nucleotide spacer of the gRNA, the sequences of the target genes were obtained from Corteva Agriscience internal database and annotated using the reference sequence of Williams 82 retrieved from Phytozome (https://phytozome.jgi.doe.gov, sequence: Glycine max Wm82.a2.v1) (Goodstein et al. 2012). The search for the 20-nucleotide spacer was performed using both Corteva proprietary software and the public genome editing guide design tool CRISPOR (Concordet and Haeussler 2018) (https://crispor.gi.ucsc.edu/, parameters: genome: Glycine max – Soybean – Phytozome 13 Wm82.a2.v1, PAM: 20bp-NGG—Sp Cas9).

### Plasmid cloning

**Fig. 1:**
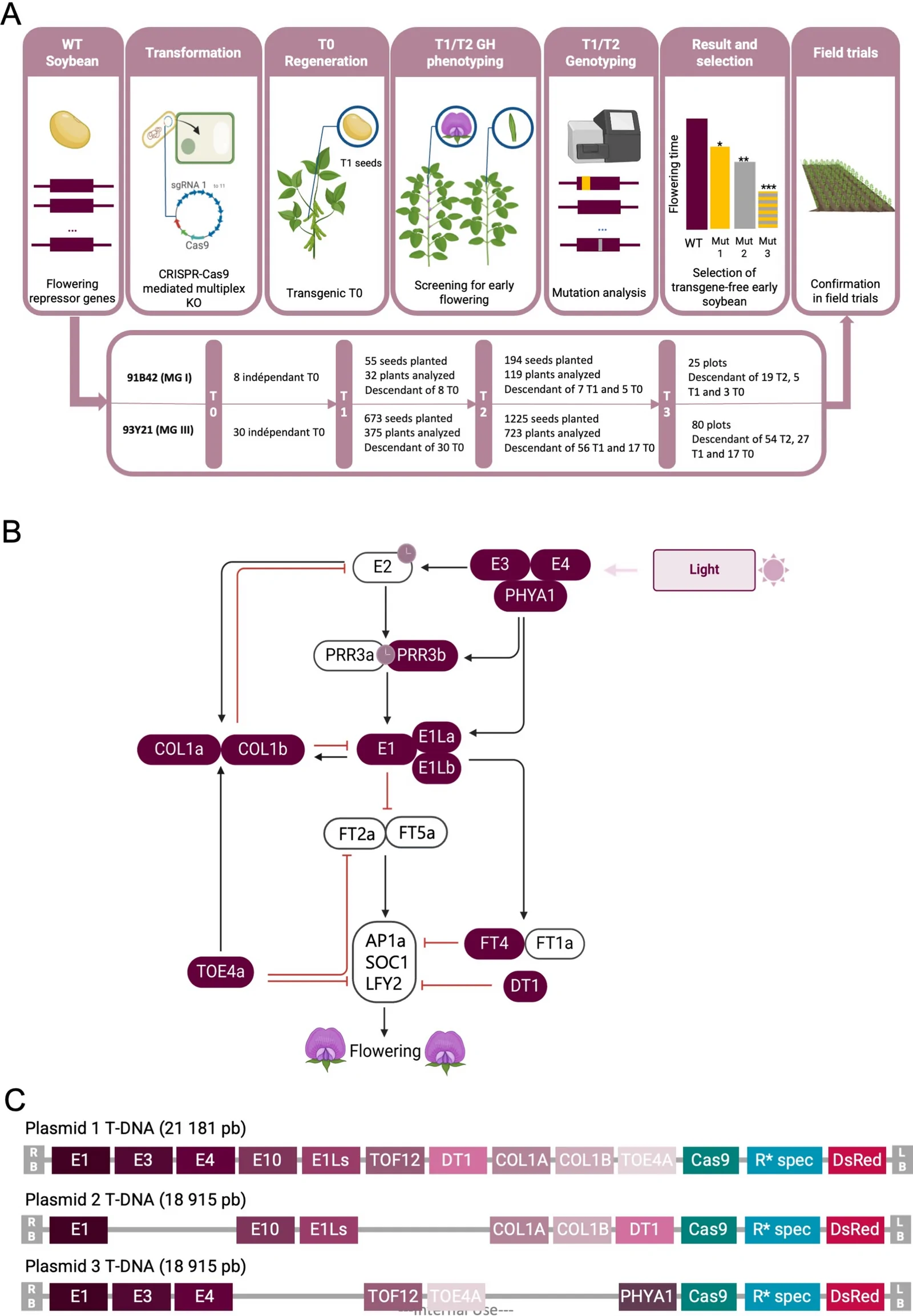
Schematic of project workflow, photoperiodic flowering pathway in soybean, and CRISPR-Cas9 multiplex knockout constructs. (**A**) General pipeline of the project. (**B**) Overview of photoperiodic regulation of flowering time in soybean. Arrows and blunt ends represent positive and negative regulation, respectively. The clock represents circadian clock proteins. PHY: Phytochrome; PRR: PSEUDO-RESPONSE REGULATORS; COL: CONSTANS-LIKE; FT: FLOWERING LOCUS T; SPL: SQUAMOSA PROMOTER BINDING PROTEIN LIKE; TOE: TARGET OF EAT; LUX: LUX ARRHYTHMO; AP1: APETALA1; SOC1: SUPPRESSOR OF OVEREXPRESSION OF CONSTANCE 1; LFY: LEAFY. Targeted flowering repressors are in white on burgundy background. (Adapted from Luo et al. 2022). Created in https://BioRender.com. (**C**) Schematic representation of T-DNA constructs used for CRISPR-Cas multiplex KO. Plasmids 1 to 3. Pink rectangles represent gRNA modules, each designed to target one specific gene except for *E1L* guide which targets two homologous genes (*E1La* and *E1Lb*). RB: T-DNA Right border, LB: T-DNA Left border, R* Spec: Spectinomycin resistance. Modules are not at scale.

Three plasmids, Plasmid 1, Plasmid 2, and Plasmid 3, were cloned for this project (Fig. 1c), each harboring a different combination of gRNAs targeting the selected genes. In addition to the gRNAs expression cassettes, each plasmid shared a common T-DNA architecture, including the Cas9, the selection marker, and the visual color marker (Figs. S8-S10).

For the gRNAs expression cassettes, the promoters *GmU6-16.2, GmU6-13.1*, or *GmU6-9.1* (Li et al. 2015) drive the constitutive expression of the gRNAs composed of the crRNA fused to the tracrRNA from *S. pyogenes* (Jinek et al. 2012; Mali et al. 2013) followed by a modified GmU6 terminator (5’-ttttttgcggcc-3’). Plasmid 1 contains ten gRNA expression cassettes targeting a total of eleven genes: *E1, E3, E4, E10, Tof12, E1La* and *E1Lb* (shared gRNA*)*, *COL1a, COL1b, TOE4a* and *DT1*. Plasmid 2 contains six gRNA modules targeting seven genes: *E1, E10, E1La* and *E1Lb, COL1a, COL1b*, and *DT1*. Plasmid 3 contains six gRNA expression cassettes targeting six genes: *E1, E3, E4, Tof12, TOE4a*, and *PHYA1*. Spacer sequences are provided in Table S3.

The Cas9 expression cassette contains a *Cas9* gene from *S. pyogenes* (Cong et al. 2013)**,** under the control of the soybean elongation factor 1A2 (*GmEF1A2*) promoter (1500 bp upstream of *Glyma.05G114900*) and the terminator of *A. thaliana* u*biquitin 10* (*AtUbi10*) gene (880 bp downstream of *AT4G05320*). To optimize its expression and to target the encoded Cas9 protein to the nucleus, the *Cas9* gene contains the second intron from the light-inducible tissue-specific protein 1 (ST-LS1) from *Solanum tuberosum* (Vancanneyt et al. 1990) and is flanked by a canola-optimized *A. thaliana* NLS (5’) and a canola-optimized *A. tumefaciens* NLS (3’) (Mysore et al. 1998).

The selection marker expression cassette is composed of a spectinomycin resistance gene *(spcN*) from *Streptomyces spectabilis* (Lyutskanova and Stoilova-Disheva 2003) under the control of *AtUbi10* promoter (917 bp upstream of *AT4G05320*) and followed by the terminator of *A. thaliana ubiquitin 14* (*AtUbi14*) gene (900 bp downstream of *AT4G02880*).

The visual marker expression cassette is composed of the *DsRed* gene from *Discosoma* sp. (Clontech monomeric 28‐kDa DsRed fluorescent protein) under the control of the soybean glycinin subunit G1 (*GmGY1*) seed promoter (Nielsen and Fischer 1989) and followed by the terminator of *A. thaliana ubiquitin 3 (AtUbi3)* gene (1087 bp downstream of *AT5G03240*).

The gRNA and Cas9 expression cassettes were synthesized by GenScript (Piscataway, NJ, USA) and subsequently cloned via Gateway cloning (Katzen 2007) into a T-DNA destination vector containing the spectinomycin selection marker and the DsRed visual marker cassettes. All plasmids were quality-controlled through deep sequencing.

### Transformation and regeneration

Soybean transformation was based on a method described previously (Cho et al. 2022). Mature seeds of 91B42 and 93Y21 genotypes were surface sterilized for 16 h using chlorine gas, produced by mixing 3.5 mL of 12 N HCl with 100 mL of commercial bleach (5.25% sodium hypochlorite) as described previously (Di et al. 1996). Then the seeds were soaked in distilled sterile water overnight at room temperature. Intact embryonic axes (EAs) were isolated using a scalpel blade. *Agrobacterium tumefaciens* strain LBA4404Thy (WO2010078445A1) containing Plasmids 1, 2 or 3, was used for transformation.

A volume of 15 mL of *Agrobacterium* suspension (OD 0.5 at 600 nm) in infection medium composed of 1/10X Gamborg B5 basal medium (G398 Phytotech Labs), 30 g/L sucrose (S5391 Sigma-Aldrich), 20 mM MES (Omnipure MES Milipore 6120), 0.25 mg/L GA3 (G7645 Sigma-Aldrich), 1.67 mg/L BAP (B3408 Sigma-Aldrich), 200 μM acetosyringone (D134406 Sigma-Aldrich), 1 mM dithiothreitol (D0632 Sigma-Aldrich), 200 µM poloxamer (Sigma Aldrich P5556) at pH 5.4 was added to the EAs, and they were plated on a 25×9×100 mm deep Petri dish (Fisherbrand FB0875711). The plates were sealed with parafilm (Cat No. 52858, ‘Parafilm M’ VWR) and then sonicated (Sonicator-VWR model 50T) for 30 s.

After sonication, EAs were incubated on a shaker at 60 rpm for 2 h at room temperature. After inoculation, excess bacterial suspension was removed and EAs were transferred to a single layer of autoclaved sterile filter paper (Cat No. 28320–020, VWR) in 25×9×100 mm Petri dish (Fisherbrand FB0875711). The plates were sealed with Micropore tape (Cat No. 1530–0, 3M, St. Paul, MN) and incubated under dim light (1–2 µE/m^2^/s), with photoperiod of 16 h at 21 °C for 3 days.

After co-cultivation, the base of each embryonic axis was embedded in solid shoot induction medium (R7100, PhytoTech Labs) containing 30 g/L sucrose (S5391 Sigma-Aldrich), 6 g/L agar (PhytoTech A296) and 25 mg/L spectinomycin (S4014 Sigma-Aldrich) as a selectable agent and 500 mg/L cefotaxime (PhytoTech C380) at pH 5.6. Shoot induction was carried out in growth room at 26 °C with a photoperiod of 16 h and a light intensity of 60–100 µE/m^2^/s.

After 4–6 weeks in selection medium, the spectinomycin-resistant shoots were cut and transferred to ½ strength MS rooting medium (M404 PhytoTech Labs) containing 15 g/L sucrose (S5391 Sigma-Aldrich), 6 g/L agar (PhytoTech A296), 10 mg/L spectinomycin (S4014 Sigma-Aldrich) and 250 mg/L cefotaxime (PhytoTech C380) for further shoot and root development.

After 2 weeks on root induction medium, transgenic soybean T_0_ plantlets were transferred to moistened Berger BM2 soil (Berger, Saint-Modeste, QC, Canada) and kept enclosed in clear plastic tray boxes until acclimatized in a growth room at 26/24 °C day/night temperatures with a photoperiod of 16-h and a light intensity of 250–350 µE/m^2^/s. Hardened plantlets were potted in 2-gallon pots containing moistened SunGro 702 and grown to maturity for harvest in a greenhouse.

### Randomization of the experiments

Randomization was achieved using R software (RStudio 2022.12.0), through the *sample* function followed by the *matrix* function. Each plant received a unique identifier, and the software generated a randomized table specifying the precise positioning of each plant in the greenhouse (Figs. S3-S4).

### Greenhouse conditions

T_1_ seeds, obtained by self-pollination of T0 plants, and T2 seeds, obtained by self-pollination of T1 plants, were studied in the same greenhouse under comparable conditions, except for the date of sowing. T_1_ seeds were sown in mid-September 2023, while T_2_ seeds were sown at the end of April 2024.

The seeds were initially sown in sand trays (Sibelco HN ½0.5). After ten days, seedlings were transplanted into 8×8 cm pots filled with potting soil (Jiffy Substrate, 60% fine black peat, 40% fine blonde peat, 4.6 kg/m^3^ lime, pH (H_2_O): 5.8 EC: 0.1) and randomly positioned on greenhouse benches. After flowering, the selected plants were transferred to 19 L pots until harvesting.

Throughout the experiment, plants were irrigated with a nutrient solution (N: 140 mg/L, P_2_O_5_: 76 mg/L, K_2_O: 231 mg/L). They were cultivated under LD conditions consisting of 16 h of light (sodium lamp), followed by 8 h of darkness, with a setpoint of 19°C and a cooling system in place.

### T_1_ and T_2_ flowering time phenotyping in greenhouse

Flowering time was assessed by calculating the number of days from sowing in sand to the R1 stage (Fehr and Caviness scale, Fehr 1977), defined as the appearance of a flower at any node on the main stem. The R1 stage was precisely identified through daily observations in the greenhouse, with monitoring conducted on weekdays (5 days out of 7) for T_1_ screening and daily observations throughout the week (7 days out of 7) for T_2_ confirmation.

### Statistical analysis

Data analysis was performed using R (RStudio 2022.12.0). For flowering time analysis, Kruskal–Wallis test was employed as a first step to evaluate whether there were statistically significant differences in flowering time or maturity among the different lines (R packages *dplyr* and *rstatix*; function *kruskal_test*). After a significant result in the Kruskal–Wallis test, post hoc pairwise comparisons were conducted using Dunn’s test (two-sided) (R packages *dplyr* and *rstatix*; function *dunn_test;* adjustment method Bonferroni). The effect of maturity group on seed weight was evaluated using a one-way Welch’s analysis of variance (Welch’s ANOVA) as the assumption of normality of residuals was met (Shapiro–Wilk test, R package *stats,* function *shapiro.test*) but not the homogeneity of variances (Levene’s test, R package *car,* function *levene.test*). Following a significant Welch’s ANOVA result (function *oneway.test*), a Games-Howell posthoc test was performed for pairwise comparisons among maturity groups (R package *rstatix,* function *games_howell_test*). A compact letter display was generated using the *cld* function from the same package to visually summarize the significant differences. For all tests, a significance level of α=0.05 was used and plots were generated using *ggplot2* package*.*

### Genotyping

To confirm and characterize targeted gene edits, T_0_, T_1_ and T_2_ plants were either genotyped by Illumina amplicon sequencing or Sanger sequencing. Both required a first step of PCR amplification. The primers and parameters used for this amplification step are summarized in Table S10-S11. For Illumina sequencing, amplicons with their adapters containing their unique index were pooled and sequenced on an Illumina MiSeq (Illumina, San Diego, CA), according to manufacturer’s instructions. After sequencing, the reads were identified by their index and binned according to their sample ID using Corteva internal analysis software. Sanger sequencing was performed by GenoScreen (GenoScreen, Lille, France). Genotyping results are summarized in Data S1-S3.

### Field trial

The field trial was planted on May 30, 2025, in Iowa, USA (MG II area). The experimental design was randomized and comprised 171 single-row plots, each 2.3 m in length with an inter-row spacing of 75 cm. Planting was executed at a rate of 60 seeds per plot, targeting a final plant density of 370,000 plants ha⁻^1^. Due to variable seed availability and germination, final plant stands ranged from 0 to 60 plants per plot. To account for the confounding effect of plant density on phenological development, plots with a final stand of fewer than 15 plants were removed from the final analysis of flowering and maturity data (18 plots). Plots were treated following standard agricultural practice for the region, with no irrigation or fertilization, a pre-emergent herbicide application on May 23rd, a post-emergent herbicide application on June 19th, and a fungicide application on August 5th. Flowering time, defined as the date on which approximately 50% of the plants in a plot displayed flowers, was scored through daily observation of the plots. Maturity, defined as the date on which approximately 95% of the plants in a plot had reached R8 stage, was scored using a similar method. At physiological maturity (R8 stage), plants were harvested and seed yield was estimated by measuring the total seed weight per plot. Oil and protein contents were analyzed nondestructively using near-infrared (NIR) spectroscopy as previously described **(**Flyckt et al. 2024). The lines tested are described in Data S3.

## Results

### Target gene selection and generation of T₀ plants

The first objective of this study was to generate novel allelic combinations for genes controlling flowering time. For this purpose, we adapted the approach known as Targeted Induced Gene Variation (TIGV**, **Nogué et al. 2016). TIGV involves the generation of allelic diversity within a defined molecular pathway and the screening of this population to identify, without prior assumptions, the optimal allele combinations that confer the desired phenotype, in this case, early flowering suited to northern latitudes (Fig. 1a).

Candidate genes whose loss of function could potentially lead to accelerated flowering were identified from the literature and selected to be targeted by CRISPR-Cas9 (Fig. 1b). Under long-day conditions, the light signal is perceived by phytochromes (E3, E4, PHYA1), which subsequently upregulate the expression of E1, E1-like genes (E1Ls), E2, GmPRR3a (Tof11), and GmPRR3b (Tof12). E1 enhances the expression of flowering repressors GmFT1a and GmFT4 while inhibiting the expression of flowering promoters GmFT2a and GmFT5a, thereby delaying the transition from the vegetative state to flowering. The induction of GmPRR3a and GmPRR3b under long-day conditions releases the inhibition of E1 expression by GmLHYs. E3 and E4 also facilitate the expression of E2. Concurrently, GmCOL1a and GmCOL1b repress the expression of GmFT2a and GmFT5a, while GmTOE4a delays flowering by inhibiting GmFT2a, GmFT5a, GmSPL3/9, and miR172, and promoting the expression of miR156, GmCOL1a, and GmCOL1b. miR172 negatively regulates its target gene, GmTOE4a, through a feedback mechanism. GmTOE4a is involved in the flowering-regulatory pathway that requires E3 and E4. Under short-day conditions, E1 expression is suppressed, removing the inhibition of GmFT2a/GmFT5a and the induction of GmFT1a/GmFT4, which strongly promotes flowering. The expression of E1/E1Ls is repressed by GmLHYs and J (GmELF3), which act additively to regulate E1 expression. J is suppressed by E3 and E4 and forms the evening complex by interacting with GmLUXs. Genes to be knocked-out were chosen based on functional evidence indicating a repressive role in soybean flowering time, including the presence of naturally occurring non-functional alleles or phenotypes resulting from gene inactivation studies. Based on these criteria, twelve genes were selected as targets for gene knockout experiments aimed at promoting early flowering phenotypes: *E1* (Xia et al. 2012), *E1La* (Dietz et al. 2021), *E1Lb* (Zhu et al. 2019), *E3* (Xu et al. 2013), *E4* (Xu et al. 2013), DT1  (Bernard 1972),  *E10*/*GmFT4* (Zhai et al. 2014), *Tof12 (GmPRR3b)* (Wang et al. 2020), *COL1a* (Wu et al. 2019), *COL1b* (Wu et al. 2019), *TOE4a* (Zhao et al. 2015) and *PHYA1* (Zhao et al. 2022) (Fig. 1b, Figs. S1-S2 and Table S1).

Cultivars 91B42 (MG I) and 93Y21 (MG III) were selected for this experiment due to their late-flowering phenotype and strong agronomic performance, with the aim of demonstrating that targeted editing could accelerate flowering time while preserving their agronomic value. The sequences of the targeted genes in both cultivars were analyzed using internal assembly data to verify the presence of functional alleles. The sequences were compared to the well-established allele status of reference genotype Williams 82. The allele combinations of each genotype for all targeted genes are detailed in Table S2. All gRNAs were designed to target a single gene, except for one gRNA that simultaneously targets *E1La* and *E1Lb,* and to minimize putative off-target cutting sites (Table S3).

Three plasmids, Plasmid 1, 2 and 3, were designed to inactivate, through NHEJ or alternative end-joining (Alt-EJ)-mediated frameshift mutation, eleven, seven and six genes, respectively, of the twelve selected soybean flowering repressors (Fig. 1c). These multiplex constructs enabled the simultaneous targeting of multiple genes within the flowering regulatory network, allowing the generation of diverse allelic combinations across T₀ lines.

*Agrobacterium*-mediated transformations were then conducted on soybean genotypes 91B42 and 93Y21, using Plasmid 1, Plasmid 2 or Plasmid 3. Functionality of the designed gRNAs was evaluated on T_0_ plantlets using Illumina sequencing of PCR amplicons. This analysis revealed that the gRNA targeting *E1La/E1Lb* was non-functional in Plasmid 2, whereas all other gRNAs displayed effective editing activity (Fig. S5). Eight independent T₀ plants from 91B42 genetic background and thirty from 93Y21 genetic background were grown to maturity in the greenhouse and self-pollinated, producing 55 and 673 T₁ seeds, respectively (Table S4).

### T1 progeny of primary CRISPR-Cas9 transformants flowers significantly earlier than wild-type (WT) control lines

To evaluate the flowering time of the progeny of T₀ plants _0_, the 673 T_1_ seeds derived from transformation of line 93Y21 (Lines #9 to #38), along with 48 93Y21 WT controls, and 36 external controls from the cultivar Williams 82 (MG III), were randomly sown in greenhouse (Fig. S3). One week after sowing, 375 T_1_ seeds had germinated similarly to the WT controls and were retained for the analysis. Seeds that failed to germinate (158) or germinated later (140), due to the transformation process, genotype, or external factors, were excluded to prevent bias in flowering time assessment. The 93Y21 control line flowered on average at 61.5 days after sowing (DAS), whereas the earliest lines #37, 14, 29, and 35, had a mean flowering time of 37 to 37.6 DAS, representing a precocity of flowering of approximately 24 days compared to the control. The population displayed a broad range of flowering times, spanning from more than three weeks earlier to two weeks earlier, one week earlier, and no difference relative to the control, demonstrating the successful generation of phenotypic diversity (Table S5). Flowering time data for the 91B42 genotype are presented in Table S6.

### Genotyping of T_1_ plants shows creation of a diversity of allelic combinations

**Fig. 2:**
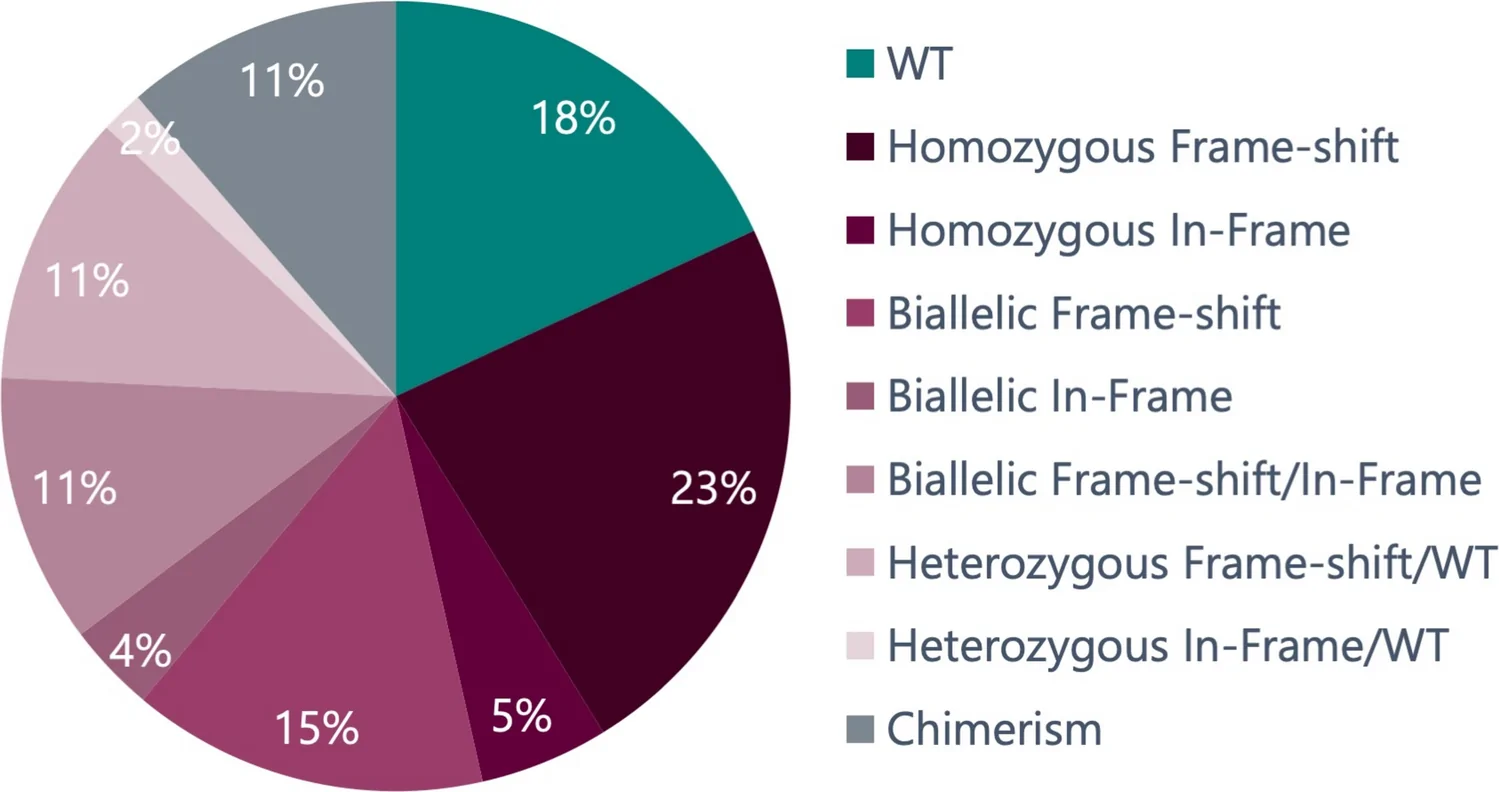
Allele distribution at targeted loci in T1 edited populations. The pie chart includes the sequencing data of all targeted loci from the 91B42 and 93Y21 T1 populations (N=2408). Data from non-transformed plants (escapes) have been removed. Homozygous refers to both copies of the gene being edited with the same allele. Biallelic refers to both copies of the gene being edited but with different alleles. Heterozygous refers to one copy of the gene being edited while the other remains wild-type.

Following phenotypic assessment of flowering time, T_1_ plants were genotyped using Illumina amplicon sequencing to evaluate the efficiency of the CRISPR-Cas9 multiplexing strategy and to establish the specific mutation combinations underlying the observed phenotypes. Sequencing revealed that 83% of the targeted loci were edited, with 28% of the loci exhibiting homozygous mutations, and 38% displaying frameshift mutations on both alleles, either as homozygous (23%) or biallelic (15%) variants, likely resulting in complete loss of gene function (Fig. 2).

The high frequency of homozygous mutations can be attributed to the same mutation occurring at both alleles, segregation following self-fertilization of heterozygous loci, or somatic homologous recombination using a mutated chromatid as a repair template (Filler Hayut et al. 2017). In contrast, 18% of loci remained homozygous wild type, and chimeric genotypes were observed at 11% of loci. These results suggest that while Cas9 activity is predominantly confined to early developmental stages, particularly in the T_0_ generation, delayed editing of wild-type alleles can occur in subsequent generations provided the CRISPR-Cas9 system is still present.

Analysis of individual loci revealed that allelic diversity varied substantially, with the number of distinct alleles created ranging from 23 at locus E3 to 73 at locus DT1 (Table S7). Across all loci, CRISPR-Cas9-induced mutations showed a strong bias toward small deletions, with 52% of alleles carrying deletions <20 bp near the predicted cutting site. Many deletions were associated with nearby microhomologies, consistent with repair via the Alt-EJ pathway. For instance, a recurrent 5-bp deletion at E1 likely involved a [GC] microhomology, and a common 27-bp deletion at COL1a appeared to result from a [GCC] microhomology (Fig. S6). However, not all deletions could be explained by the presence of microhomology, consistent with reports that Alt-EJ can also mediate repair independently of pre-existing microhomologies (Mara et al. 2019). Single base-pair indels accounted for approximately 10% of alleles, reflecting the tendency of CRISPR-Cas9-induced repair to generate single-nucleotide insertions that duplicate nearby bases via classical non-homologous end joining (C-NHEJ) repair pathway (Pallaseni et al. 2024) (Fig. S6). Insertions of more than 1 bp represented 14% of alleles, with the largest insertion being 112 bp. Several sequences could not be reliably identified using BLAST, likely due to their short length, whereas others matched soybean genomic sequences, including segments from the target gene, distant nuclear loci, or chloroplast genome. Finally, approximately one-third of mutations were in-frame, consistent with a random distribution of indels and no obvious repair bias toward frameshift or in-frame mutations. These results indicate that the CRISPR-Cas9 multiplexing strategy successfully generated a broad spectrum of mutations across multiple loci, achieving the intended goal of creating allelic diversity in soybean flowering time regulatory pathway.

### Phenotyping of T_2_ plants confirms inheritance of CRISPR-induced early flowering trait

**Fig. 3:**
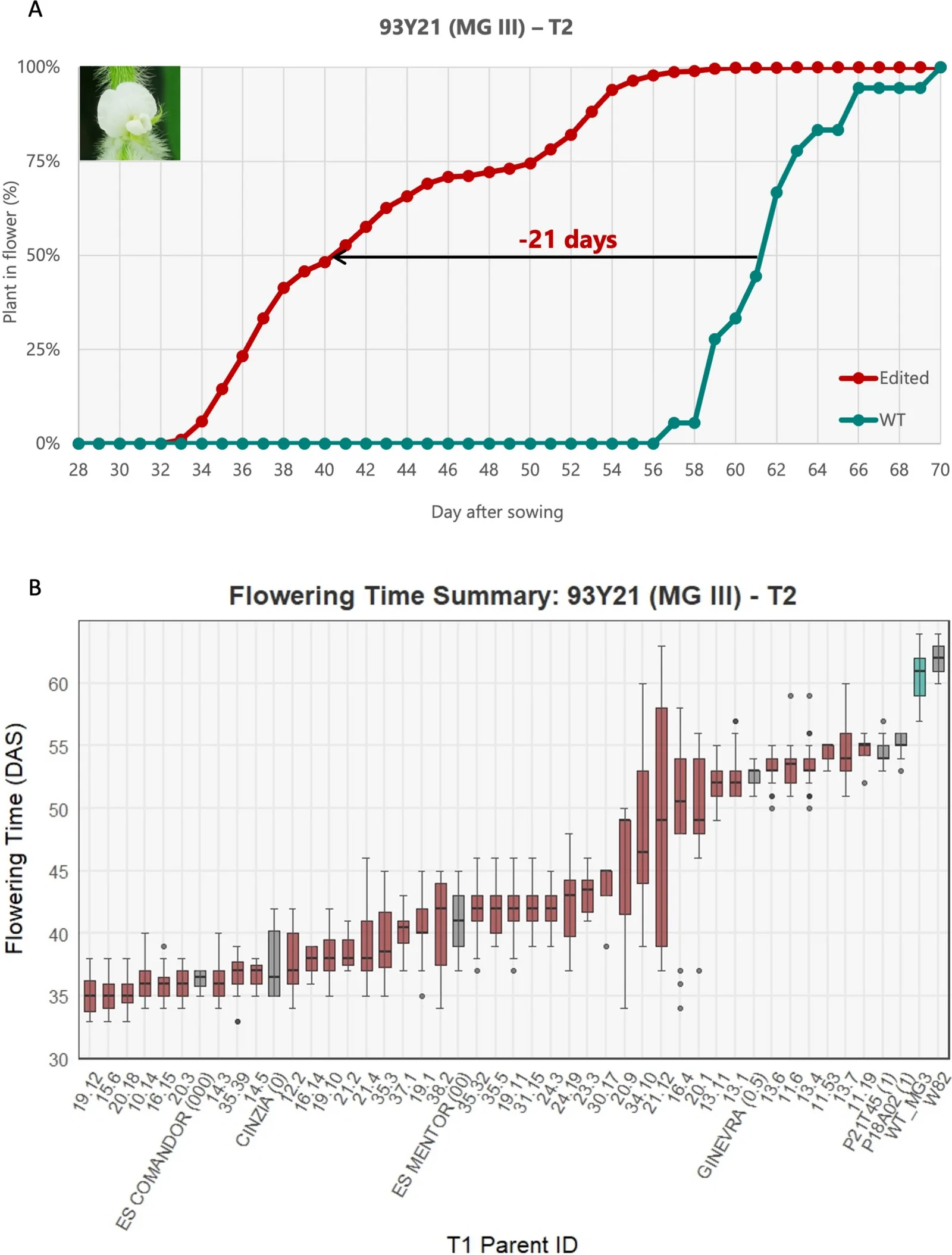
T_2_ flowering time analysis for 93Y21 population. (**A**) Cumulative percentage of individual T_2_ plants reaching R1 as a function of the number of days after sowing. The analysis includes 723 edited T_2_ plants and 18 WT plants. (**B**) Flowering time distributions of individual T_2_ progeny families derived from the indicated T_1_ plants. Each boxplot represents the T_2_ plants derived from one T1 parent. Edited T2 progeny families are in red, wild type in blue, and external commercial control in grey. The numbers in brackets correspond to the maturity groups of the commercial controls. DAS: Days after sowing.

To confirm the inheritance of the early flowering time observed in T_1_, T_2_ seeds harvested from a diverse set of self-pollinated early flowering T_1_ lines, together with WT and external commercial controls were randomly sown under greenhouse conditions (Fig. S4). Commercial controls, ranging from MG 000 to MG III, were used to position the gene-edited lines on the MG scale. For 93Y21 genotype, out of the 1225 T_2_ seeds originating from 39 T_1_ plants and the 60 93Y21 controls, 723 had germinated properly and were retained for analysis. Figure 3a summarizes flowering at the level of these individual T2 plants, whereas Fig. 3b classifies these plants according to their T1 parental origin. Thus, Fig. 3b illustrates segregation and within-family variation that is not apparent from the population-level distribution shown in Fig. 3a. The analysis of 93Y21 T_2_ edited population revealed a continuous variation in flowering time across different lines, contrasting with the uniform flowering time observed in WT controls, and underscoring the successful generation of flowering time diversity (Fig. 3a).

Notably, the earliest edited lines, such as #19.12, #15.6, and #20.18, flowered 35 days after sowing (DAS) compared with 62 DAS for the WT, representing a 27-day advancement (Fig. 3b, Table S8). This shift is slightly earlier than that observed in their T_1_ parental lines and precedes the flowering time of commercial cultivars such as ES Comandor (MG 000). Other lines, such as #30.17 and #13.6, showed more moderate precocity of one to two weeks, collectively reflecting a high degree of phenotypic diversity within the edited population (Fig. 3b, Table S8). While most lines exhibited uniform flowering behavior, a few lines (e.g., #20.9, #21.12, #34.10) showed intra-line variability, likely reflecting segregation from heterozygous T_1_ parents (Fig. 3b).

Overall, this second phenotyping analysis confirms the successful creation of soybean lines showing a range of flowering times compared to their respective wild types, with flowering accelerated by up to 27 days in the 93Y21 population. Results for the 91B42 population are available in Fig. S7 and Table S9.

### E1 loss of function is the major driver of early flowering, while additional edits further modify flowering time

Combined analysis of flowering-time and genotype data highlighted a strong association between E1 loss of function and early flowering under the tested long-day conditions. T1 lines retaining WT or in-frame E1 alleles generally flowered later than lines carrying frameshift E1 alleles. Conversely, E1 frameshift genotypes combined with additional mutations frequently displayed the earliest flowering phenotypes. In the T2 generation, lines carrying an apparent E1 loss-of-function genotype alone, such as #38.2 and #24.9, flowered approximately 20–22 days earlier than the WT control, whereas lines carrying E1 loss of function together with three or four additional edited loci flowered approximately one week earlier than lines lacking E1 alone (Data S1 and S2).

Because the multiplex population did not contain balanced representation of all possible allele combinations, the dataset does not permit independent estimation of the effect of every targeted gene or formal testing of all pairwise interactions. The observations should therefore be interpreted as genotype/phenotype associations that identify candidate contributors to flowering-time variation rather than as a complete genetic dissection of the pathway.

Within the available genotype combinations, frameshift mutations in E3 and E4 were associated with earlier flowering in some genetic backgrounds, consistent with their previously reported roles in flowering-time regulation. However, because these mutations occurred in combination with other edits, their individual contributions cannot be quantified independently in the present population. Similarly, Tof12 and PHYA1 mutations did not produce an obvious additional flowering-time shift among the combinations represented here, although the available genotypes do not permit us to exclude context-dependent effects.

### Validation of early flowering under field conditions

To validate the flowering time phenotypes observed in the T_1_ and T_2_ generations under greenhouse conditions, 72 edited lines were evaluated in field trials. These lines originated from 20 and 52 edited T_2_ plants derived from the 91B42 (MG I) and 93Y21 (MG III) genotypes, respectively. Prior to field evaluation, all selected lines were confirmed to be T-DNA–free by SbS (Southern-by-Sequencing) (Zastrow‐Hayes et al. 2015), and only these verified lines were advanced to field testing. Field trials were conducted in May 2025 within a maturity group II region. The corresponding WT 91B42 and 93Y21 lines, along with nine Corteva Agriscience commercial or pre-commercial controls ranging from MG 000 to MG 0, were included as comparators.

**Fig. 4:**
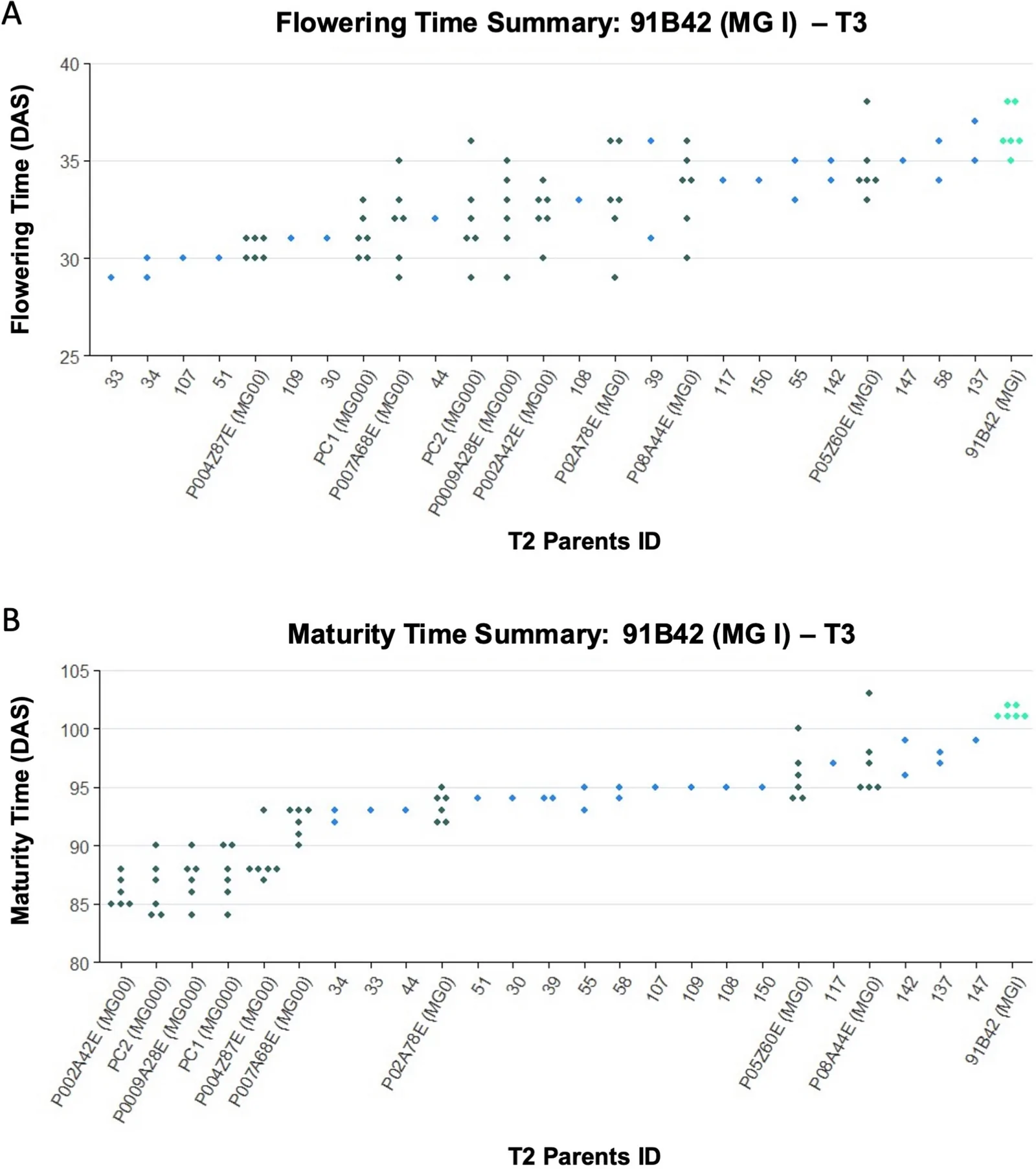
Flowering and maturity time of edited lines in the T_3_ field trial (Genotype 91B42). (**A**-**B**) Flowering time and maturity time obtained during T_3_ field trial for genotype 91B42. Each dot corresponds to one plot. Edited lines are in blue, wild type in light green, and external commercial controls in dark green. The numbers in brackets correspond to the maturity groups of the commercial controls. DAS: Days after sowing.

**Fig. 5:**
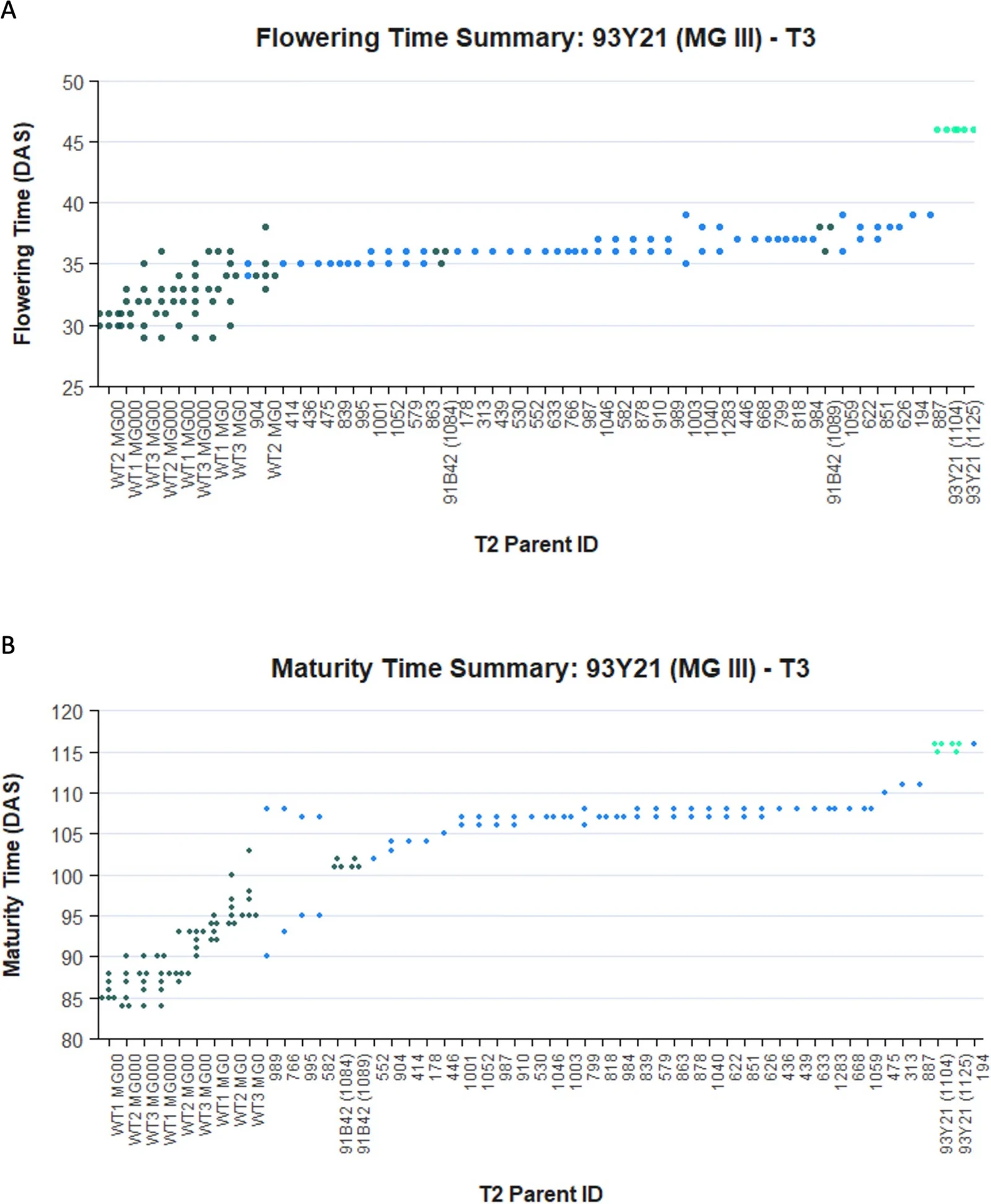
Flowering and maturity time of edited lines in the T_3_ field trial (Genotype 93Y21). (**A**-**B**) Flowering time and maturity time obtained during T_3_ field trial for genotype 93Y21. Each dot corresponds to one plot. Edited lines are in blue, wild type in light green, and external commercial controls in dark green. The numbers in brackets correspond to the maturity groups of the commercial controls. DAS: Days after sowing. PC: Pre-commercial.

The field trial confirmed that the edited lines flowered earlier than their respective WT controls; however, the magnitude of flowering-time advancement was reduced relative to that observed under greenhouse conditions. In the field, the earliest 91B42 edited lines flowered approximately one week earlier than the WT (Fig. 4a), compared with up to 24 days earlier under greenhouse conditions. Similarly, most 93Y21 edited lines flowered approximately ten days earlier than the WT in the field (Fig. 5a), whereas flowering occurred up to 27 days earlier in the greenhouse. Notably, in contrast to greenhouse observations, plots containing 93Y21 edited lines did not flower earlier than the MG 00–MG 000 commercial controls in the field, but instead exhibited flowering times comparable to the MG I control.

### Earlier flowering drives earlier maturity in soybean genotypes

**Fig. 6:**
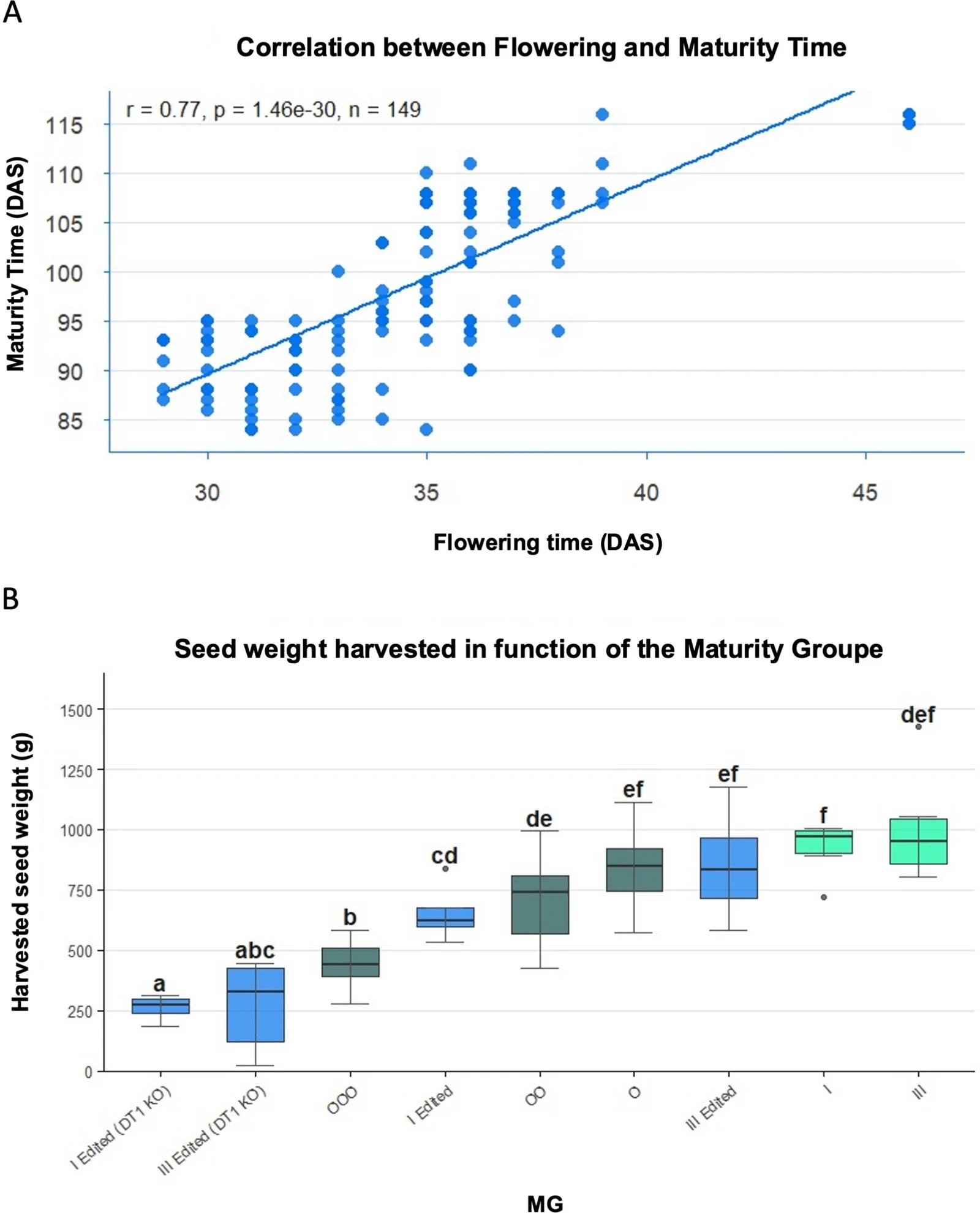
Association between flowering time, maturity time, and yield across soybean maturity groups. (**A**) Earlier flowering is correlated with earlier maturity in soybean. Scatter plot of maturity time as a function of flowering time for all edited and control genotypes. Each point represents an individual experimental plot. The blue line indicates the best-fit linear regression model (Pearson’s r = 0.77, p < 0.001). The coefficient of determination (R^2^) indicates that flowering time explains approximately 59% of the variance in maturity time. (**B**) Seed harvested per plot as a function of the Maturity Group. Boxplots show the distribution of harvested seed weight (g) for T_3_ plots tested in the field, grouped by their respective Maturity Group (MG). Edited lines derived from parental lines 91B42 (MG I) and 93Y21 (MG III) were further sub-grouped based on their DT1 genotype, as DT1 knockout reduces plant size, with low pod more difficult to harvest, hypothesized to reduce final yield. A one-way Welch’s ANOVA was performed to test for significant differences in mean seed weight among the groups. The assumption of normality of residuals was met (Shapiro–Wilk test, p-value = 0.517) but not the assumption of homogeneity of variances (Levene’s test, p-value = 0.01). The Welch’s ANOVA highlights significant yield differences depending on the Maturity Group (F value = 32.05; p-value=1.818e−15). Pairwise comparisons were conducted using a Games-Howell post hoc test. Groups that do not share a letter are significantly different from each other (p-value < 0.05).

Maturity assessment of early-flowering lines showed an average advancement of one week for 91B42 edited lines (Fig. 4b) and two weeks for 93Y21 edited lines (Fig. 5b), resulting in maturity times comparable to those of MG 0 and MG I controls, respectively. Correlation analysis between flowering time and maturity time yielded a Pearson correlation coefficient of *r* = 0.77 (p = 1.46 × 10⁻^3^⁰), indicating a strong and statistically significant positive association. However, flowering time accounted for about 59% of the observed variation in maturity time (R^2^ ≈ 0.59), suggesting that additional factors also contribute to the regulation of maturity (Fig. 6a).

Field data further supported our findings on the pivotal role of the *E1* gene in controlling soybean maturation. The 93Y21 WT reached maturity after an average of 116 days, whereas lines carrying a single *E1* knockout matured earlier, with an average of 108 days. This effect was amplified in the multiplexed edit lines, in which combining the *E1* knockout with edits in three or four additional target genes reduced the average time to maturity to 102 days (Data S3).

Agronomic performance of the edited lines was evaluated by measuring seed yield (g) from individual field plots. To minimize bias due to uneven plant establishment, plots containing fewer than 40 established plants were excluded from the analysis (57 of 172 total plots). As expected, commercial control varieties exhibited a clear trend of increasing yield with later maturity groups, reflecting the typical yield penalty associated with shorter growing cycle. A major limitation was observed in plots with edited *DT1* knockout lines, which exhibited a severely dwarfed phenotype under field conditions (Fig. S11). While the determinate growth habit was apparent in the greenhouse, plants in the field were extremely short, with pods positioned close to the ground, complicating harvest and resulting in low yields. Plots planted with *DT1* knockout lines yielded an average of 262 g (n=7) and 268 g (n=5) for the lines coming from 91B42 and 93Y21, respectively (Fig. 6b). Despite the limited sample size, the consistently low yields suggest that these lines are likely unsuitable for commercial deployment under current conditions. In contrast, edited lines retaining an indeterminate growth phenotype (*DT1* WT) showed more promising agronomic performance. The 91B42 (MG I) edited lines yielded 644 g (n=8), showing no significant yield difference from the MG 00 (700 g, n=18) commercial variety (Fig. 6b).

Similarly, 93Y21 (MG III) edited lines produced a mean yield of 843 g (n=28), with no significant difference compared to MG 00 (700 g, n=18), MG 0 (833 g, n=18), MG I (925 g, n=6), or MG III (1004 g, n=6) commercial varieties (Fig. 6b). Together, these results demonstrate that the gene editing strategy employed in this study can generate early-flowering and earlier maturing soybean lines without incurring a severe yield penalty, achieving yields comparable to or only slightly lower than commercial varieties of similar maturity groups.

To assess potential pleiotropic effects of edits of the flowering time genes on seed quality, compositional analyses on mature seeds were conducted using Near-Infrared (NIR) spectroscopy. Protein and oil contents of the edited lines were comparable to those of the commercial controls. Oil content ranged from 18.1% to 21.4% in the edited lines, within the 17.7% to 21.6% range observed in controls. Similarly, protein content in edited lines ranged from 33.1% to 39.5%, closely matching the 33.1% to 38.8% range of the controls (Data S3). These results indicate that accelerating flowering and maturity through targeted gene editing did not adversely affect critical seed composition traits.

## Discussion

### Multiplex gene editing creates extensive allelic diversity in soybean

Multiplex CRISPR-Cas9 editing has previously been successfully applied in soybean; however, the number of targeted genes has typically been limited to three to six per experiment, with some studies utilizing two gRNAs per gene, resulting in plasmids containing up to eight gRNAs (Li et al. 2021; Duan et al. 2021; Lin et al. 2023; Chen et al. 2024; Liu et al. 2025). In another study, pooled transformations using multiple vectors, each carrying a different gRNA, generated T_0_ plants with up to five targeted genes (Bai et al. 2020). In these studies, the primary objective was to obtain knockouts for the targeted genes. Our study represents an application of multiplex CRISPR-Cas9 targeting up to eleven genes simultaneously in soybean, with the objective of generating allelic diversity within a defined flowering-time pathway rather than recovering a single predefined genotype.

Editing rate analyses across the targeted loci confirmed the effectiveness of this strategy. Several plants were successfully edited at all targeted loci, demonstrating the robustness of the CRISPR–Cas9 system. As intended, other lines displayed a combination of wild-type and edited alleles, generating a broad spectrum of frameshift, in-frame, and WT configurations. Across the T_1_ populations, 83% of targeted loci were edited, with 28% carrying homozygous mutations and 38% carrying frameshift mutations on both alleles, either as homozygous or biallelic variants. Although not all theoretically possible combinations could be recovered, given the number of possible allelic configurations relative to the number of plants analyzed, the strategy generated substantial genetic diversity across the targeted flowering time pathway.

This diversity is particularly relevant for quantitative traits such as flowering time, for which the optimal phenotype may depend on the combination and relative effects of several genes. Rather than restricting the experiment to a predetermined combination of complete knockouts, the generation of multiple allelic states provides an opportunity to identify combinations associated with different phenotypic intensities. These newly created alleles could significantly enrich the pool of genetic variants available to breeders (Tsubokura et al. 2014; Miladinović et al. 2018), who currently rely on a relatively limited number of alleles at certain loci, such as *e1-as* (Dietz et al. 2021). Other loci, like *COL1s*, rarely used in soybean breeding due to limited identified natural variation (Awal Khan et al. 2022), could now be considered. The genetic variability introduced in this study generates a large range of phenotypic outcomes, among which certain variants may confer selective advantages under specific conditions. This diversity is particularly valuable when interactions within a given gene network are not fully understood and when the optimal combination of mutations required to achieve a desired phenotype is unknown. In such contexts, the allelic diversity generated by multiplexed CRISPR-Cas9 approach and the inherent variability of NHEJ/Alt-EJ-mediated repair outcomes can be advantageous. It may facilitate the discovery of novel, beneficial gene interactions and phenotypes that would likely remain undetected using more focused, single-gene approaches. In the long run, this strategy could be highly valuable for creating phenotypic variation within genetic pathways associated with other complex polygenic traits, such as drought tolerance or nutrient use efficiency (Monfort et al. 2025).

Analysis of mutations across the targeted loci revealed that most edits consisted of small deletions or single base pair insertions duplicating an adjacent nucleotide, a mutational signature characteristic of C-NHEJ or Alt-NHEJ repair pathways. These patterns are consistent with CRISPR-Cas9 outcomes reported in other organisms (Leenay et al. 2019; Pallaseni et al. 2024). Larger insertions (> 2 bp) were also observed, originating either from sequences too short to trace by BLAST or from the soybean genome itself. The predominance of small indels among early-flowering edited lines could be advantageous in the context of the evolving global regulatory landscape for genome-edited crops. In many countries, plants carrying such edits and lacking exogenous DNA from transformation vectors are exempted from the biotechnology or GMO regulation and are classified as conventional crops. This principle is also reflected in the recently adopted European framework for certain New Genomic Techniques (NGTs) (https://eur-lex.europa.eu/eli/reg/2026/1388/oj/eng). Consequently, the bias of NHEJ DNA repair toward generating small mutations may facilitate future commercialization of early-flowering soybean lines in Europe, further enhancing the value of CRISPR-Cas9 as a tool for introducing targeted genetic diversity.

### A game-changing flowering time advancement in only two generations

Phenotypic evaluation of the edited populations revealed substantial reduction in flowering time. Under greenhouse long-day conditions, the earliest edited lines exhibited a flowering time advancement of up to 25 days in 91B42 (MG I) background and 27 days in 93Y21 (MG III) background. When evaluated under field conditions in an MG II geographic area, these advancements translated to approximately one week and two weeks earlier flowering, respectively. Thus, the magnitude of the phenotype was strongly dependent on the environmental conditions, but the direction of the flowering-time effect was maintained.

The field experiment also illustrates the importance of evaluating flowering-time phenotypes under agronomically relevant conditions. In contrast to the greenhouse experiment, the earliest 93Y21-derived edited lines did not flower earlier than the MG 00–MG 000 commercial controls in the field, but instead displayed flowering times comparable to the MG I control. Similarly, the differences between the edited lines and commercial controls did not always allow an unambiguous assignment to a particular maturity group. Therefore, the present data demonstrate the generation of earlier-flowering phenotypes rather than establishing definitive maturity-group classifications.

The accelerated flowering observed in the edited lines was accompanied by earlier maturity. The average maturity advancement was approximately one week for edited 91B42 lines and two weeks for edited 93Y21 lines. Flowering time and maturity were strongly correlated, with a Pearson correlation coefficient of 0.77; however, flowering time explained only approximately 59% of the observed variation in maturity. This indicates that although flowering time is a useful indicator of maturity, additional genetic and environmental factors contribute to the duration between flowering and maturity. Consequently, direct field assessment of maturity remains essential when evaluating early-flowering genotypes for breeding purposes.

Except for one line (line #1.1), which displayed severe dwarfism, a high rate of flower abortion, and abnormal pod development, greenhouse observations did not reveal any other plants with obvious undesirable phenotypes affecting non-target traits. Line #1.1’s strong phenotype could potentially be attributed to the simultaneous targeted knockout of *E1* and its homologs. Similar reductions in plant height have been previously observed when both *E1* and *E1L*s genes are inactivated (Liu et al. 2022), consistent with reports of genetic compensation by *E1L* observed in the absence of functional *E1* (Wan et al. 2022; Fang et al. 2024).

Although the determinate growth habit induced by *DT1* knockout was expected (Bernard 1972) and already evident in greenhouse conditions, its impact was significantly exacerbated in the field. These plants exhibited severe dwarfism, reduced pod set, and poor harvestability, rendering them unsuitable for agricultural use under the tested conditions. This highlights the critical importance of field trials for soybean maturity evaluation, as deleterious traits may only manifest under realistic environmental constraints. In contrast, edited lines retaining an indeterminate phenotype maintained favorable agronomic characteristics and yield performance.

Overall, this study demonstrates the feasibility of converting a late-flowering soybean variety with a desirable genetic background into an earlier flowering one within a remarkably short timeframe, thereby complementing previous studies showing that inactivating repressors such as *E1* and its homologs can advance flowering (Liu et al. 2022; Gao et al. 2024). However, the magnitude and agronomic consequences of the resulting phenotypes are strongly environment- and genotype-dependent, highlighting the importance of subsequent field evaluation.

### Early flowering soybean: *E1* inactivation, essential but not sufficient

Although dissecting the contribution of each individual gene was challenging, the central role of *E1* inactivation in driving early flowering and maturity was clearly confirmed. However, further reductions in flowering time were achieved through the additive effects of inactivating additional target genes, enabling finer optimization. Notably, even in the absence of *E1* inactivation, knockouts of other genes produced intermediate early-flowering phenotypes.

These observations indicate that E1 is a major driver of early flowering under the long-day conditions used here, but that its inactivation does not fully determine the flowering-time phenotype. Additional mutations can further modify flowering time, resulting in a gradient of phenotypes rather than a single discrete shift. This is consistent with the complex genetic regulation of flowering in soybean and illustrates the potential advantage of generating allelic combinations across several components of the pathway.

The available genotype/phenotype relationships also provide indications regarding the relative contribution of individual targets. Within the combinations recovered in this study, E3 and E4 frameshift genotypes were associated with earlier flowering in several lines, suggesting that these loci may contribute additional effects to the E1-dependent phenotype. In contrast, Tof12 and PHYA1 mutations did not produce an obvious additional flowering-time shift in the combinations represented in the present populations. These observations should, however, be interpreted cautiously. Because the multiplex population was not designed as a balanced factorial experiment, the different target combinations were not equally represented, and many possible combinations were not recovered. Consequently, the present data do not permit independent quantitative estimates of the effect of every targeted gene or formal testing of all possible genetic interactions.

The genotype/phenotype relationships generated here should therefore be considered as evidence for candidate contributions to flowering-time variation rather than as a complete genetic dissection of the soybean flowering pathway. Nevertheless, the results provide useful information for the next iteration of the approach. The strong association between E1 loss of function and early flowering identifies E1 as a major entry point for generating substantial flowering-time shifts, while the additional phenotypic effects observed in multiplex-edited genotypes indicate that other loci can subsequently be used to fine-tune the magnitude of this response.

### Genotype/phenotype relationships inform subsequent flowering-time breeding

An important outcome of the multiplex strategy is that it generated not only early-flowering plants, but also a range of flowering-time phenotypes associated with different combinations of edited loci. This genotype/phenotype diversity can be useful in two complementary ways.

First, the resulting lines constitute a targeted source of flowering-time variation within elite soybean genetic backgrounds. Lines differing by only a few days in flowering time may be particularly valuable for evaluating the relationship between flowering time, maturity, and agronomic performance across contrasting environments. Rather than selecting only the earliest genotype, breeders could therefore use the available gradient of phenotypes to identify combinations that provide an appropriate flowering-time shift without introducing undesirable developmental or architectural effects.

Second, the genotype/phenotype information generated by the population can help prioritize combinations for subsequent breeding or editing experiments. The present results suggest that a strategy combining the major effect of E1 loss of function with additional, more moderate effects from other loci could generate a series of intermediate phenotypes. Such combinations could be particularly useful when the desired adaptation requires a specific flowering-time window rather than the maximum possible advancement.

This approach may complement conventional breeding based on naturally occurring loss-of-function alleles. Where suitable natural alleles exist, the genotype combinations identified here could provide hypotheses for allele pyramiding; where natural variation is limited, targeted editing provides an alternative route to generate comparable functional variation directly in an elite background.

At the same time, the present population should not be considered a complete diversity panel for environmental adaptation. Its value as a breeding resource will depend on subsequent multi-environment evaluation of the selected genotypes. In particular, the difference between greenhouse and field flowering-time responses demonstrates that genotype effects cannot be interpreted independently of environmental conditions.

### Field validation and perspective

Field trials were essential for validating greenhouse findings and assessing maturity, yield, and seed composition. The field trial results suggest the successful generation of early-flowering and early-maturing lines adapted to field conditions, while retaining desirable agronomic traits that could be leveraged in future breeding programs. While flowering time served as a useful proxy, the incomplete correlation with maturity underscores the need for direct field evaluation. Limited seed availability restricted testing to a single site, which was suitable for MG II but suboptimal for very early lines, potentially limiting precise maturity classification and hindering a full understanding of the performance potential of the earliest lines. These field results therefore represent an initial proof of concept, and wider multi-location trials across high-latitude European environments will be required to fully assess agronomic performance and environmental stability (Khaipho-Burch et al. 2024).

The availability of lines differing by only a few days in flowering time but otherwise genetically identical opens opportunities for designing varietal mixtures. Such mixtures could enhance crop resilience under increasingly variable climatic conditions by buffering flowering time across a population and reducing the risk of yield loss due to unpredictable weather events. In parallel, the genotype/phenotype relationships generated in this study could help guide the selection of specific flowering-time allele combinations for subsequent breeding or genome-editing experiments. Together, these applications could contribute to the development of soybean material better suited to a broader range of production environments, although the agronomic value and environmental stability of the generated lines remain to be established through further field testing.

## Conclusions

Our multiplex CRISPR–Cas9 strategy generated substantial allelic and flowering-time diversity within two elite soybean backgrounds. E1 loss of function was a major driver of early flowering, while additional mutations further modified flowering time. Selected edited lines retained earlier flowering and maturity under field conditions without apparent adverse effects on seed composition. These lines provide promising breeding material and a genotype/phenotype resource for further selection and targeted editing. Multi-location and multi-year trials will be required to assess environmental stability and agronomic value.

## Supplementary Information

Below is the link to the electronic supplementary material.

Supplementary file 1 Document S1. Figures S1–S10.

Supplementary file 2 Data S1. T1 greenhouse data.

Supplementary file 3 Data S2. T2 greenhouse data.

Supplementary file 4 Data S3. T3 field trial data.

Supplementary file 5 Document Tables S1–S10.

## Data Availability

All data supporting the findings of this study are available within the paper and its Supplementary Information.
