# Supplementary material for: Targeted allelic diversification of flowering genes via CRISPR-Cas enables the development of multiple early-flowering soybean lines
Source: Theor Appl Genet. 2026 Sep 29;139(10):286. doi: 10.1007/s00122-026-05400-9 (PMC13623812; doi:10.1007/s00122-026-05400-9)
Supplement: Supplementary file 1 [file 122_2026_5400_MOESM1_ESM.pdf]

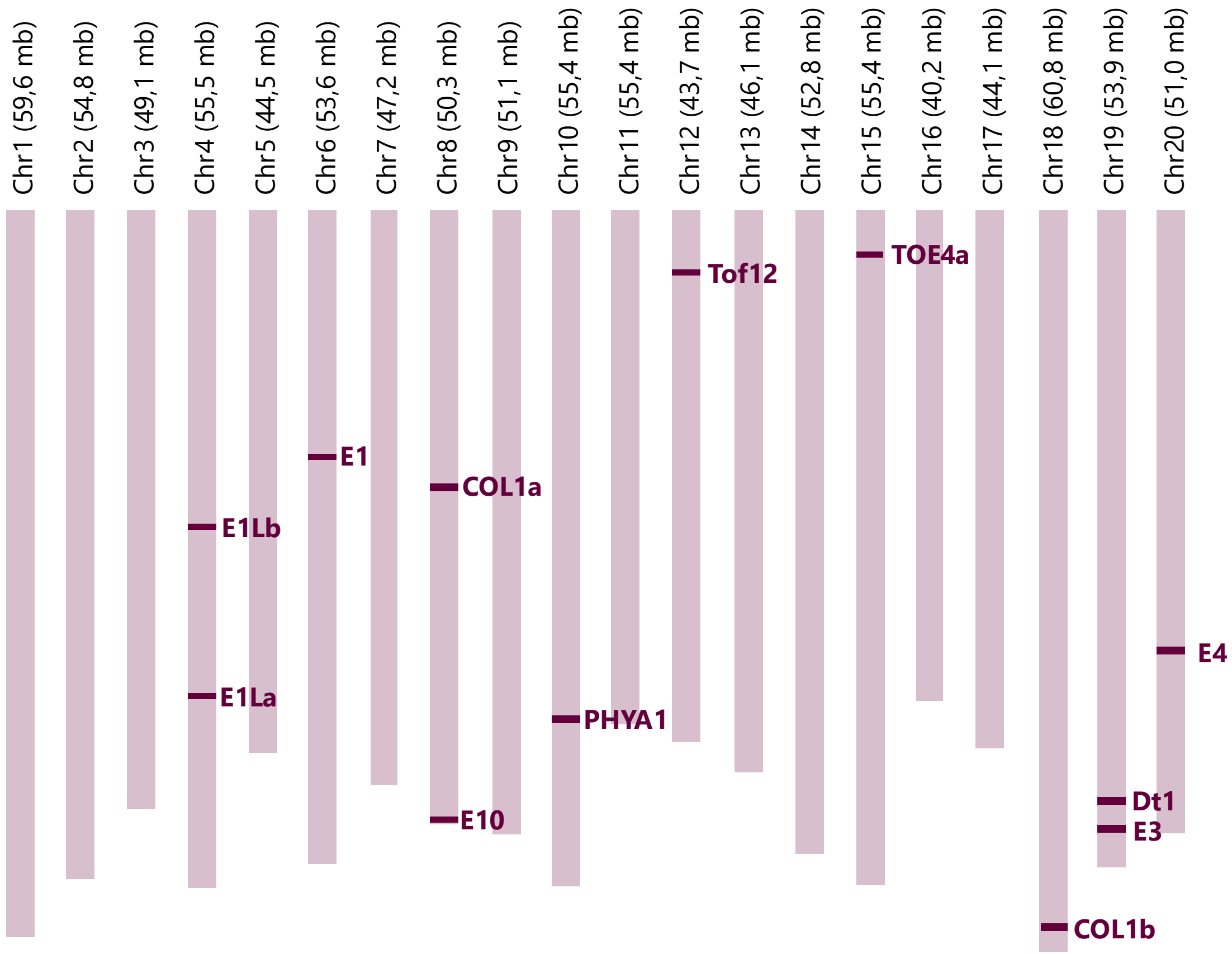


**Figure S1. Position of the targeted genes on the reference genome of variety William 82.**
Bars represent the 20 chromosomes in soybean and are scaled to chromosome sizes. (based on Phytozome G.max Wm82.a6.v1 assembly).

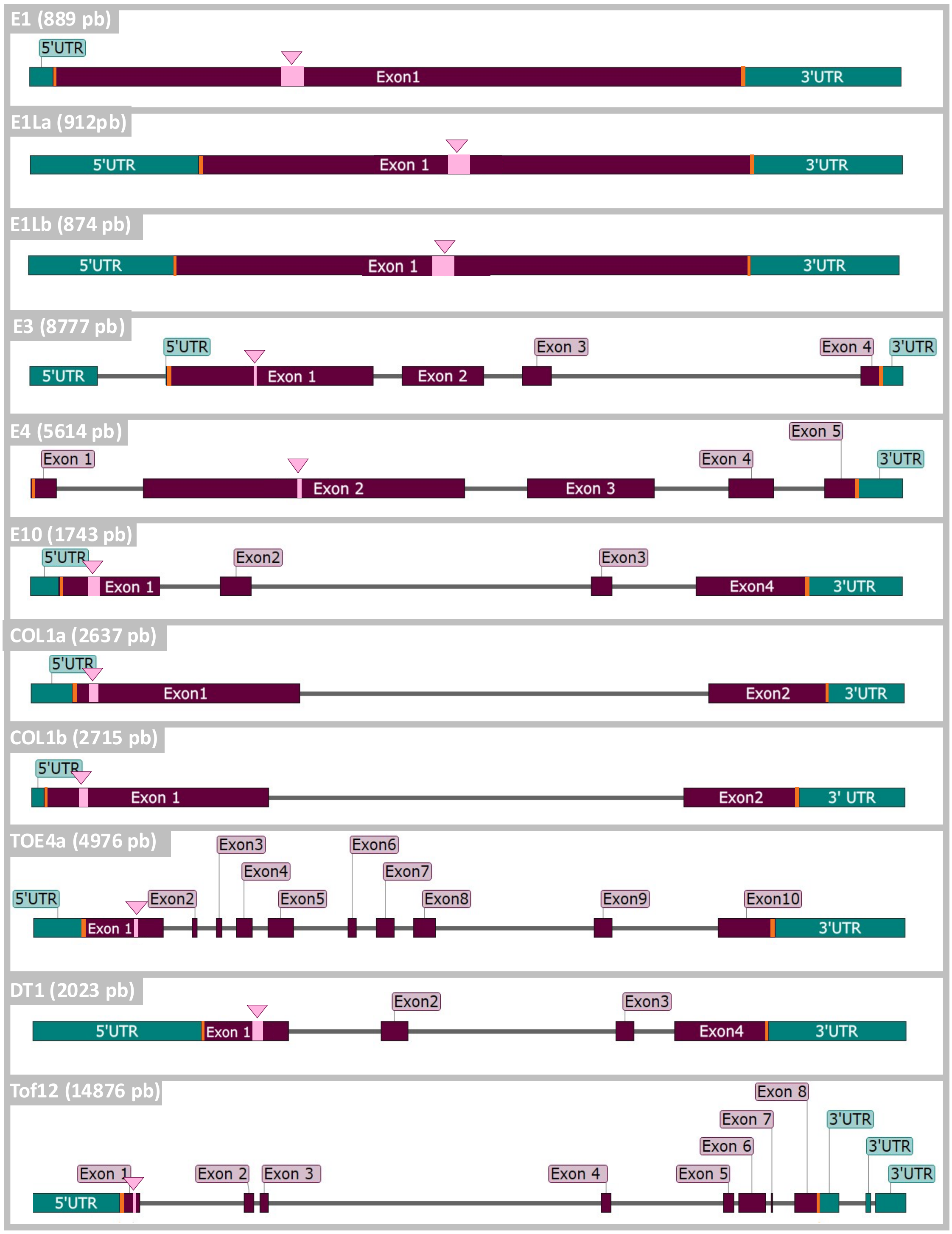


**Figure S2. Structure of the targeted genes in genotype 91B42.**
Targeted sequences are in pink and the cutting sites are indicated with the pink triangle, start and stop codons are in orange. Representation not at scale between different genes. Annotation based on Phytozome G.max Wm82.a4.v1 assembly.

| | | | | | | | | | | | | | | | |
|---|---|---|---|---|---|---|---|---|---|---|---|---|---|---|---|
| 40.9 | 28.7 | 31.11 | 30.7 | 33.12 | 3.6 | 31.15 | 10.13 | 35.4 | 25.3 | 9.5 | 21.12 | 32.1 | 21.2 | 6.13 | 39.32 |
| 19.28 | 24.3 | 25.9 | 4.8 | 2.3 | 11.42 | 13.6 | 1.1 | 3.12 | 19.27 | 39.8 | 10.5 | 11.3 | 13.9 | 11.37 | 24.18 |
| 33.4 | 35.16 | 16.1 | 24.33 | 33.6 | 39.33 | 10.6 | 40.25 | 25.13 | 11.41 | 23.2 | 27.22 | 35.23 | 34.5 | 24.15 | 24.19 |
| 11.48 | 39.25 | 33.9 | 25.30 | 33.65 | 24.30 | 16.15 | 33.2 | 34.23 | 24.25 | 34.42 | 27.27 | 20.7 | 26.17 | 40.6 | 11.38 |
| 24.1 | 4.13 | 19.21 | 6.12 | 34.30 | 19.16 | 35.30 | 26.7 | 33.68 | 26.31 | 4.4 | 13.1 | 28.23 | 24.36 | 26.44 | 27.12 |

| | | | | | | | | | | | | | | | |
|---|---|---|---|---|---|---|---|---|---|---|---|---|---|---|---|
| 40.2 | 20.12 | 25.16 | 26.13 | 19.12 | 34.24 | 9.3 | 26.9 | 27.4 | 33.66 | 30.16 | 31.10 | 19.30 | 20.21 | 25.15 | 39.24 |
| 20.4 | 35.41 | 17.7 | 33.10 | 15.7 | 40.29 | 28.19 | 35.34 | 26.51 | 19.11 | 31.2 | 24.28 | 34.45 | 2.5 | 12.5 | 20.20 |
| 30.2 | 36.2 | 34.37 | 29.3 | 19.4 | 20.3 | 10.19 | 20.6 | 39.5 | 14.4 | 40.14 | 26.26 | 11.33 | 11.22 | 35.8 | 9.4 |
| 11.26 | 39.12 | 35.35 | 33.8 | 31.18 | 11.18 | 19.34 | 15.2 | 20.10 | 33.36 | 13.5 | 24.9 | 10.17 | 26.8 | 40.3 | 33.26 |
| 26.28 | 27.21 | 17.13 | 33.39 | 38.2 | 10.7 | 16.8 | 34.1 | 27.8 | 19.22 | 10.15 | 33.58 | 24.26 | 30.18 | 34.12 | 17.17 |

| | | | | | | | | | | | | | | | |
|---|---|---|---|---|---|---|---|---|---|---|---|---|---|---|---|
| 40.17 | 26.6 | 33.64 | 20.11 | 16.19 | 33.49 | 25.27 | 19.5 | 14.2 | 30.11 | 26.53 | 28.15 | 33.38 | 8.2 | 37.1 | 39.30 |
| 33.61 | 4.2 | 34.31 | 20.18 | 24.4 | 40.10 | 34.20 | 34.21 | 28.10 | 6.2 | 35.29 | 25.20 | 28.22 | 10.14 | 28.9 | 34.18 |
| 28.28 | 14.1 | 33.29 | 15.8 | 33.14 | 34.55 | 11.10 | 25.17 | 39.22 | 15.15 | 40.28 | 15.17 | 24.12 | 15.12 | 11.12 | 11.28 |
| 33.51 | 39.18 | 31.16 | 10.4 | 19.29 | 30.12 | 20.24 | 30.5 | 27.17 | 34.7 | 25.26 | 11.34 | 20.9 | 26.55 | 40.31 | 25.25 |
| 25.31 | 27.6 | 34.47 | 29.4 | 17.15 | 35.22 | 20.23 | 26.36 | 33.69 | 34.44 | 33.30 | 26.10 | 10.3 | 19.10 | 27.16 | 19.6 |

| | | | | | | | | | | | | | | | |
|---|---|---|---|---|---|---|---|---|---|---|---|---|---|---|---|
| 40.18 | 10.2 | 28.3 | 16.4 | 28.6 | 26.19 | 21.10 | 6.11 | 11.20 | 6.6 | 24.14 | 12.1 | 30.17 | 11.40 | 33.11 | 39.9 |
| 18.1 | 31.8 | 34.25 | 11.50 | 28.21 | 11.21 | 33.21 | 35.18 | 34.6 | 11.30 | 39.29 | 19.20 | 17.10 | 36.1 | 16.6 | 11.43 |
| 11.47 | 33.24 | 4.10 | 34.49 | 3.10 | 39.13 | 11.5 | 40.13 | 25.24 | 30.10 | 15.5 | 26.48 | 25.28 | 26.54 | 33.25 | 16.17 |
| 19.24 | 39.17 | 13.10 | 26.38 | 35.14 | 11.11 | 25.18 | 37.5 | 31.9 | 25.7 | 26.27 | 15.9 | 33.50 | 35.15 | 40.1 | 3.9 |
| 26.30 | 33.52 | 26.52 | 31.6 | 26.46 | 26.16 | 11.44 | 10.21 | 13.11 | 6.5 | 24.35 | 12.6 | 35.24 | 17.9 | 10.1 | 35.20 |

| | | | | | | | | | | | | | | | |
|---|---|---|---|---|---|---|---|---|---|---|---|---|---|---|---|
| 40.20 | 30.3 | 19.25 | 27.18 | 34.40 | 35.9 | 33.40 | 26.35 | 19.8 | 35.38 | 37.6 | 29.2 | 33.19 | 34.8 | 24.34 | 39.19 |
| 31.5 | 21.5 | 26.12 | 11.2 | 7.1 | 40.19 | 19.9 | 33.53 | 25.12 | 16.13 | 39.34 | 27.14 | 33.35 | 25.22 | 27.26 | 35.36 |
| 30.9 | 26.42 | 24.6 | 26.45 | 19.2 | 39.28 | 28.27 | 15.16 | 28.20 | 10.18 | 40.11 | 35.43 | 35.32 | 31.13 | 25.19 | 25.14 |
| 35.6 | 39.10 | 25.1 | 6.8 | 29.1 | 34.62 | 33.56 | 26.61 | 19.7 | 28.4 | 20.2 | 19.23 | 26.41 | 33.7 | 40.36 | 34.26 |
| 10.10 | 33.22 | 28.17 | 33.55 | 28.29 | 33.62 | 26.24 | 23.1 | 17.18 | 11.19 | 6.4 | 34.58 | 30.6 | 26.33 | 20.5 | 24.8 |

| | | | | | | | | | | | | | | | |
|---|---|---|---|---|---|---|---|---|---|---|---|---|---|---|---|
| 40.21 | 28.1 | 34.34 | 34.4 | 10.22 | 11.6 | 34.15 | 28.8 | 35.44 | 27.10 | 27.3 | 17.12 | 26.4 | 2.4 | 20.13 | 39.11 |
| 35.7 | 3.7 | 12.3 | 34.27 | 21.7 | 40.22 | 28.26 | 11.54 | 28.14 | 27.2 | 39.23 | 25.35 | 35.31 | 34.22 | 16.14 | 11.9 |
| 28.2 | 34.35 | 16.2 | 20.8 | 16.3 | 39.21 | 21.6 | 30.8 | 35.42 | 33.20 | 40.27 | 17.4 | 6.7 | 26.50 | 11.27 | 33.48 |
| 28.31 | 39.3 | 26.29 | 33.59 | 25.4 | 20.17 | 22.1 | 11.39 | 25.36 | 35.21 | 33.31 | 26.43 | 26.11 | 34.43 | 40.23 | 28.13 |
| 33.54 | 11.31 | 26.47 | 31.17 | 35.19 | 2.1 | 26.1 | 20.19 | 19.13 | 34.16 | 33.63 | 25.37 | 27.24 | 4.1 | 14.6 | 33.67 |

| | | | | | | | | | | | | | | | |
|---|---|---|---|---|---|---|---|---|---|---|---|---|---|---|---|
| 40.12 | 34.50 | 20.1 | 11.1 | 16.11 | 26.40 | 35.5 | 4.14 | 5.3 | 33.13 | 3.5 | 24.31 | 19.36 | 11.45 | 5.1 | 39.27 |
| 26.57 | 27.9 | 21.3 | 19.17 | 19.14 | 19.19 | 10.11 | 16.5 | 30.14 | 34.19 | 39.14 | 34.53 | 21.4 | 19.26 | 11.24 | 17.14 |
| 26.34 | 11.15 | 16.18 | 28.5 | 38.3 | 39.7 | 35.3 | 40.4 | 14.3 | 37.2 | 2.2 | 6.3 | 32.2 | 34.54 | 27.15 | 34.2 |
| 8.1 | 39.36 | 20.22 | 13.8 | 26.59 | 16.12 | 28.30 | 24.22 | 26.15 | 6.14 | 24.24 | 4.7 | 17.3 | 11.35 | 40.5 | 26.21 |
| 26.14 | 24.32 | 33.17 | 25.29 | 4.6 | 27.20 | 4.12 | 24.16 | 35.33 | 13.4 | 19.31 | 4.11 | 19.33 | 27.19 | 31.19 | 26.37 |

| | | | | | | | | | | | | | | | |
|---|---|---|---|---|---|---|---|---|---|---|---|---|---|---|---|
| 40.33 | 36.3 | 3.2 | 35.46 | 38.1 | 24.23 | 12.2 | 35.37 | 6.10 | 11.13 | 26.23 | 19.15 | 17.11 | 5.2 | 21.11 | 39.6 |
| 30.15 | 28.16 | 21.1 | 25.2 | 25.34 | 40.35 | 33.42 | 11.29 | 35.40 | 20.15 | 4.9 | 33.45 | 33.34 | 34.29 | 31.3 | 10.9 |
| 4.5 | 25.33 | 35.39 | 33.3 | 24.11 | 33.27 | 24.7 | 35.11 | 39.16 | 21.9 | 40.32 | 34.10 | 35.12 | 11.51 | 30.13 | 33.16 |
| 33.37 | 39.15 | 34.9 | 10.24 | 11.14 | 34.38 | 21.8 | 11.46 | 31.1 | 9.1 | 24.20 | 11.52 | 35.28 | 35.10 | 40.16 | 25.5 |
| 4.15 | 28.18 | 37.3 | 30.20 | 25.23 | 33.57 | 17.19 | 10.16 | 15.6 | 35.2 | 24.17 | 34.51 | 26.56 | 26.62 | 25.21 | 26.60 |

| | | | | | | | | | | | | | | | |
|---|---|---|---|---|---|---|---|---|---|---|---|---|---|---|---|
| 40.8 | 11.17 | 34.17 | 21.13 | 35.25 | 28.25 | 11.4 | 34.56 | 34.52 | 34.13 | 27.1 | 34.48 | 20.16 | 19.32 | 37.4 | 39.26 |
| 26.39 | 27.25 | 33.33 | 16.10 | 27.23 | 40.34 | 3.3 | 34.36 | 26.32 | 34.60 | 24.10 | 34.33 | 24.29 | 33.44 | 34.11 | 11.49 |
| 12.4 | 11.53 | 25.10 | 30.19 | 15.3 | 27.7 | 34.32 | 16.16 | 39.1 | 13.2 | 40.24 | 24.13 | 34.28 | 8.3 | 6.1 | 11.7 |
| 15.14 | 39.31 | 33.15 | 17.2 | 31.7 | 35.1 | 3.4 | 20.14 | 6.9 | 11.25 | 11.32 | 28.11 | 19.35 | 25.8 | 40.7 | 9.2 |
| 13.3 | 4.3 | 34.46 | 10.20 | 26.49 | 33.47 | 26.2 | 26.3 | 24.5 | 10.12 | 3.1 | 11.55 | 13.7 | 15.11 | 34.3 | 28.24 |

| | | | | | | | | | | | | | | | |
|---|---|---|---|---|---|---|---|---|---|---|---|---|---|---|---|
| 40.26 | 10.8 | 30.4 | 27.13 | 34.57 | 24.27 | 34.41 | 33.46 | 11.56 | 16.7 | 34.14 | 37.8 | 11.36 | 33.41 | 35.27 | 39.35 |
| 37.7 | 33.28 | 26.58 | 31.4 | 17.6 | 15.13 | 35.17 | 25.6 | 24.21 | 2.6 | 39.20 | 28.33 | 27.11 | 31.14 | 24.2 | 26.5 |
| 3.11 | 3.8 | 33.43 | 26.20 | 27.5 | 39.2 | 15.1 | 40.15 | 19.18 | 25.32 | 16.9 | 17.5 | 19.1 | 28.32 | 33.18 | 35.45 |
| 33.5 | 39.4 | 16.20 | 35.13 | 34.59 | 30.1 | 10.23 | 33.23 | 15.4 | 26.25 | 15.10 | 34.61 | 34.39 | 25.11 | 40.30 | 26.22 |
| 23.3 | 11.16 | 17.8 | 17.1 | 33.1 | 26.18 | 17.16 | 33.60 | 11.23 | 31.12 | 11.8 | 14.5 | 35.26 | 33.32 | 28.12 | 19.3 |

Door

**Figure S3. Sowing plan for T1 generation.**
The plan was randomized (except for wild-type plants) with no block due to an unbalanced experimentation. Control groups are in blue with group 39 and 40 corresponding to 91B42 and 93Y21 respectively.

| | | | | | | | | | | | | | | | | | | | | | | | | | | | | | | | |
|---|---|---|---|---|---|---|---|---|---|---|---|---|---|---|---|---|---|---|---|---|---|---|---|---|---|---|---|---|---|---|---|
| 1325 | 196 | 713 | 870 | 111 | 692 | 1165 | 1102 | 849 | 1106 | 636 | 880 | 1258 | 128 | 915 | 833 | 1375 | 46 | 721 | 311 | 370 | 583 | 760 | 1346 | 1224 | 1427 | 1374 | 438 | 511 | 37 | 1115 | 809 |
| 151 | 875 | 43 | 889 | 461 | 366 | 742 | 542 | 1412 | 1160 | 1154 | 1061 | 1259 | 942 | 737 | 963 | 146 | 10 | 1304 | 879 | 612 | 682 | 1401 | 3 | 926 | 422 | 139 | 1289 | 1080 | 285 | 1042 | 1372 |
| 878 | 733 | 1367 | 1186 | 1112 | 1063 | 1034 | 1137 | 135 | 648 | 1051 | 972 | 556 | 1098 | 1434 | 1331 | 819 | 8 | 1251 | 946 | 1152 | 130 | 168 | 470 | 1048 | 655 | 584 | 952 | 120 | 105 | 641 | 778 |
| 677 | 1246 | 61 | 100 | 290 | 483 | 477 | 843 | 900 | 818 | 936 | 594 | 627 | 35 | 64 | 348 | 1265 | 803 | 59 | 486 | 314 | 717 | 795 | 600 | 352 | 347 | 1056 | 1027 | 1294 | 1185 | 145 | 1206 |
| 562 | 113 | 1183 | 924 | 65 | 1333 | 749 | 176 | 1337 | 1049 | 868 | 66 | 1301 | 776 | 1261 | 68 | 36 | 1142 | 24 | 469 | 838 | 364 | 1296 | 666 | 281 | 837 | 253 | 22 | 1366 | 1030 | 738 | 133 |
| 1308 | 798 | 599 | 714 | 1231 | 994 | 82 | 1364 | 553 | 456 | 669 | 784 | 162 | 706 | 291 | 1197 | 679 | 1161 | 662 | 910 | 1050 | 529 | 1198 | 684 | 829 | 2 | 1254 | 201 | 271 | 467 | 150 | 48 |
| 598 | 1339 | 1022 | 991 | 322 | 1091 | 1280 | 724 | 626 | 854 | 602 | 400 | 731 | 442 | 852 | 460 | 979 | 510 | 1201 | 325 | 426 | 323 | 1219 | 1209 | 299 | 409 | 180 | 813 | 178 | 1035 | 916 | 1109 |
| 1066 | 1383 | 1284 | 478 | 501 | 1045 | 610 | 977 | 632 | 691 | 1257 | 74 | 921 | 1232 | 198 | 528 | 1006 | 260 | 1324 | 101 | 446 | 581 | 676 | 919 | 1230 | 508 | 1088 | 1038 | 688 | 1121 | 185 | 635 |
| 1220 | 1033 | 720 | 248 | 1216 | 1018 | 76 | 1014 | 410 | 418 | 242 | 273 | 771 | 119 | 286 | 1297 | 1376 | 1282 | 545 | 618 | 31 | 831 | 988 | 1348 | 25 | 170 | 425 | 430 | 735 | 309 | 1285 | 156 |
| 828 | 1225 | 1191 | 1 | 1157 | 1433 | 1336 | 20 | 1194 | 238 | 319 | 1290 | 412 | 1153 | 783 | 399 | 491 | 772 | 337 | 268 | 9 | 1275 | 1023 | 884 | 671 | 377 | 507 | 955 | 1127 | 297 | 961 | 1226 |
| 525 | 664 | 1139 | 995 | 1338 | 356 | 306 | 708 | 1350 | 851 | 298 | 1052 | 788 | 1311 | 1413 | 480 | 964 | 214 | 179 | 1410 | 380 | 1380 | 901 | 830 | 474 | 1387 | 390 | 353 | 1249 | 484 | 317 | 782 |
| 1095 | 414 | 1193 | 475 | 932 | 1373 | 1078 | 705 | 989 | 643 | 494 | 848 | 850 | 940 | 821 | 1431 | 1371 | 663 | 499 | 137 | 1313 | 182 | 417 | 704 | 332 | 420 | 466 | 709 | 874 | 1187 | 611 | 639 |
| 1279 | 1158 | 272 | 1293 | 233 | 762 | 904 | 613 | 1188 | 453 | 1089 | 13 | 1104 | 255 | 1388 | 766 | 668 | 660 | 944 | 97 | 152 | 1316 | 1181 | 254 | 1322 | 746 | 195 | 1330 | 1100 | 619 | 761 | 169 |
| 575 | 15 | 1092 | 1094 | 1276 | 1321 | 1184 | 215 | 973 | 1329 | 1163 | 806 | 394 | 596 | 270 | 465 | 1190 | 999 | 540 | 719 | 997 | 318 | 33 | 73 | 1079 | 522 | 485 | 723 | 1242 | 966 | 891 | 1256 |
| 1395 | 755 | 841 | 72 | 1025 | 244 | 1057 | 210 | 876 | 996 | 741 | 49 | 847 | 381 | 689 | 1266 | 1074 | 736 | 836 | 184 | 164 | 262 | 1031 | 487 | 374 | 362 | 775 | 609 | 1218 | 1270 | 976 | 396 |
| 535 | 239 | 710 | 1399 | 1117 | 1009 | 793 | 558 | 428 | 341 | 893 | 883 | 450 | 368 | 1228 | 110 | 437 | 715 | 80 | 820 | 79 | 235 | 642 | 920 | 1418 | 823 | 172 | 539 | 1111 | 232 | 567 | 1070 |
| 959 | 960 | 1352 | 1419 | 429 | 1229 | 240 | 132 | 1347 | 589 | 816 | 802 | 142 | 857 | 930 | 707 | 236 | 1093 | 779 | 218 | 764 | 427 | 191 | 1233 | 1268 | 607 | 1096 | 378 | 1272 | 1288 | 1039 | 1151 |
| 971 | 1075 | 949 | 1404 | 52 | 839 | 826 | 116 | 1155 | 12 | 550 | 452 | 1207 | 582 | 578 | 345 | 495 | 1013 | 846 | 570 | 899 | 695 | 163 | 1028 | 551 | 308 | 1277 | 887 | 908 | 1342 | 1087 | 379 |
| 1414 | 537 | 165 | 579 | 26 | 757 | 832 | 791 | 980 | 1320 | 32 | 1377 | 1196 | 1140 | 700 | 1240 | 905 | 1162 | 333 | 894 | 1425 | 70 | 698 | 67 | 862 | 1060 | 122 | 703 | 42 | 188 | 463 | 241 |
| 1306 | 335 | 824 | 1357 | 1420 | 1072 | 1319 | 40 | 136 | 842 | 365 | 855 | 497 | 444 | 1164 | 740 | 186 | 1351 | 1264 | 1173 | 279 | 1314 | 886 | 811 | 92 | 197 | 867 | 796 | 301 | 1043 | 1391 | 166 |
| 561 | 560 | 482 | 1409 | 1145 | 312 | 1273 | 568 | 1244 | 277 | 94 | 431 | 1015 | 216 | 1169 | 464 | 801 | 1136 | 173 | 1212 | 903 | 1172 | 680 | 769 | 161 | 27 | 822 | 435 | 906 | 1147 | 247 | 1167 |
| 590 | 1044 | 148 | 1195 | 339 | 845 | 1110 | 134 | 633 | 344 | 1200 | 1426 | 23 | 981 | 359 | 1405 | 658 | 275 | 983 | 29 | 199 | 536 | 580 | 227 | 81 | 777 | 1090 | 1141 | 1392 | 114 | 1040 | 630 |
| 623 | 421 | 604 | 434 | 768 | 307 | 526 | 1012 | 371 | 815 | 576 | 86 | 175 | 1124 | 1054 | 316 | 384 | 917 | 974 | 117 | 1064 | 888 | 1192 | 58 | 765 | 310 | 351 | 1069 | 393 | 805 | 640 | 423 |
| 651 | 1436 | 661 | 207 | 962 | 716 | 355 | 935 | 882 | 126 | 781 | 864 | 109 | 647 | 354 | 320 | 102 | 673 | 503 | 1149 | 386 | 230 | 4 | 1386 | 357 | 471 | 812 | 810 | 7 | 1202 | 329 | 514 |
| 750 | 911 | 943 | 785 | 1237 | 984 | 557 | 504 | 125 | 1118 | 237 | 1135 | 330 | 160 | 1081 | 773 | 631 | 790 | 967 | 521 | 1402 | 748 | 284 | 1179 | 1119 | 200 | 953 | 1406 | 457 | 159 | 524 | 727 |
| 407 | 220 | 1381 | 869 | 154 | 219 | 294 | 71 | 259 | 563 | 519 | 1369 | 1327 | 276 | 789 | 958 | 1340 | 1168 | 492 | 360 | 653 | 88 | 591 | 615 | 574 | 1300 | 96 | 1344 | 343 | 734 | 1116 | 295 |
| 340 | 605 | 451 | 1215 | 302 | 628 | 104 | 506 | 28 | 245 | 448 | 1400 | 449 | 1065 | 622 | 115 | 1024 | 758 | 767 | 872 | 118 | 672 | 657 | 1073 | 167 | 1134 | 476 | 1241 | 346 | 890 | 923 | 411 |
| 595 | 1021 | 385 | 701 | 177 | 1385 | 1130 | 18 | 1307 | 1305 | 807 | 1292 | 897 | 1390 | 107 | 90 | 1046 | 213 | 1159 | 1019 | 753 | 520 | 39 | 1223 | 181 | 1435 | 266 | 405 | 683 | 1309 | 1011 | 646 |
| 678 | 458 | 1295 | 21 | 1144 | 577 | 47 | 1208 | 1177 | 518 | 5 | 441 | 447 | 1370 | 1437 | 951 | 759 | 593 | 565 | 382 | 592 | 388 | 728 | 1047 | 634 | 538 | 305 | 93 | 770 | 1175 | 1082 | 149 |
| 292 | 63 | 108 | 1260 | 1017 | 1150 | 1360 | 881 | 313 | 222 | 87 | 1432 | 744 | 670 | 711 | 725 | 1008 | 1213 | 895 | 462 | 752 | 571 | 250 | 440 | 397 | 652 | 993 | 1122 | 246 | 1123 | 398 | 685 |
| 1353 | 204 | 554 | 856 | 1247 | 228 | 69 | 859 | 937 | 490 | 56 | 564 | 871 | 267 | 1026 | 1286 | 1341 | 1416 | 865 | 121 | 205 | 1120 | 1037 | 597 | 402 | 1302 | 1174 | 763 | 860 | 625 | 931 | 694 |
| 722 | 712 | 274 | 985 | 934 | 369 | 693 | 106 | 687 | 89 | 193 | 287 | 1394 | 730 | 505 | 1126 | 718 | 1055 | 1363 | 637 | 45 | 555 | 243 | 265 | 933 | 234 | 57 | 1189 | 1408 | 1398 | 543 | 1166 |
| 1328 | 774 | 1263 | 1007 | 1428 | 1227 | 827 | 1424 | 413 | 877 | 606 | 1250 | 1415 | 1287 | 547 | 1125 | 212 | 1269 | 614 | 513 | 1020 | 1361 | 321 | 1255 | 569 | 157 | 439 | 1368 | 123 | 509 | 975 | 644 |
| 229 | 656 | 638 | 902 | 209 | 419 | 621 | 443 | 194 | 1156 | 1267 | 787 | 1071 | 454 | 804 | 939 | 1103 | 153 | 190 | 699 | 326 | 552 | 515 | 586 | 667 | 1010 | 794 | 99 | 1214 | 616 | 251 | 395 |
| 1062 | 55 | 533 | 1131 | 1440 | 406 | 947 | 124 | 1358 | 189 | 601 | 1407 | 1108 | 1362 | 645 | 155 | 143 | 1146 | 493 | 675 | 572 | 548 | 1278 | 331 | 531 | 225 | 350 | 909 | 44 | 16 | 954 | 1411 |
| 969 | 144 | 208 | 334 | 690 | 925 | 416 | 950 | 1138 | 986 | 1343 | 1099 | 992 | 263 | 858 | 1000 | 1393 | 998 | 14 | 987 | 252 | 726 | 928 | 75 | 131 | 1252 | 17 | 315 | 223 | 1114 | 665 | 754 |
| 747 | 1148 | 697 | 516 | 436 | 408 | 1332 | 624 | 1001 | 50 | 1291 | 141 | 1077 | 502 | 1429 | 140 | 929 | 800 | 853 | 261 | 1129 | 1365 | 1107 | 1238 | 1354 | 912 | 217 | 60 | 91 | 473 | 649 | 415 |
| 982 | 78 | 174 | 293 | 1205 | 358 | 1204 | 1430 | 1182 | 34 | 303 | 530 | 1084 | 603 | 957 | 289 | 1133 | 914 | 129 | 866 | 1299 | 907 | 1002 | 1170 | 620 | 304 | 54 | 559 | 835 | 617 | 1239 | 376 |
| 11 | 817 | 1323 | 527 | 1283 | 459 | 278 | 192 | 517 | 19 | 51 | 1356 | 1016 | 751 | 1085 | 1271 | 387 | 256 | 77 | 62 | 481 | 336 | 338 | 938 | 1421 | 498 | 1355 | 799 | 1345 | 1379 | 403 | 861 |
| 948 | 112 | 756 | 1423 | 780 | 1005 | 445 | 138 | 38 | 608 | 1067 | 1303 | 541 | 455 | 549 | 327 | 1262 | 1029 | 1132 | 585 | 328 | 918 | 282 | 588 | 432 | 978 | 1298 | 183 | 103 | 1217 | 945 | 913 |
| 231 | 732 | 1349 | 566 | 1236 | 956 | 1245 | 389 | 1222 | 1378 | 1326 | 1171 | 1180 | 674 | 743 | 372 | 361 | 1312 | 896 | 892 | 1438 | 30 | 1003 | 922 | 1235 | 500 | 203 | 1310 | 797 | 85 | 532 | 1059 |
| 523 | 970 | 1113 | 1439 | 834 | 965 | 127 | 98 | 863 | 147 | 1143 | 1417 | 479 | 1234 | 84 | 534 | 512 | 1359 | 363 | 468 | 1105 | 968 | 324 | 342 | 206 | 729 | 814 | 401 | 53 | 41 | 258 | 1397 |
| 1083 | 825 | 1076 | 283 | 95 | 1221 | 1128 | 211 | 1281 | 488 | 654 | 249 | 375 | 1036 | 1211 | 898 | 496 | 696 | 280 | 1403 | 1210 | 1041 | 1334 | 404 | 745 | 224 | 264 | 257 | 300 | 489 | 808 | 990 |
| 171 | 1004 | 221 | 1199 | 1389 | 686 | 83 | 202 | 433 | 844 | 383 | 1253 | 1086 | 544 | 1317 | 587 | 472 | 1101 | 702 | 296 | 1384 | 1335 | 650 | 1097 | 885 | 226 | 1176 | 1248 | 629 | 927 | 840 | 739 |
| 786 | 1315 | 573 | 1396 | 391 | 1382 | 269 | 1178 | 1053 | 187 | 1068 | 681 | 288 | 424 | 373 | 392 | 1032 | 1318 | 158 | 349 | 1203 | 6 | 659 | 873 | 367 | 941 | 792 | 1058 | 1243 | 1422 | 1274 | 546 |

**Figure S4. Sowing plan for T2 generation.**
The plan was randomized with no block due to an unbalanced experimentation.

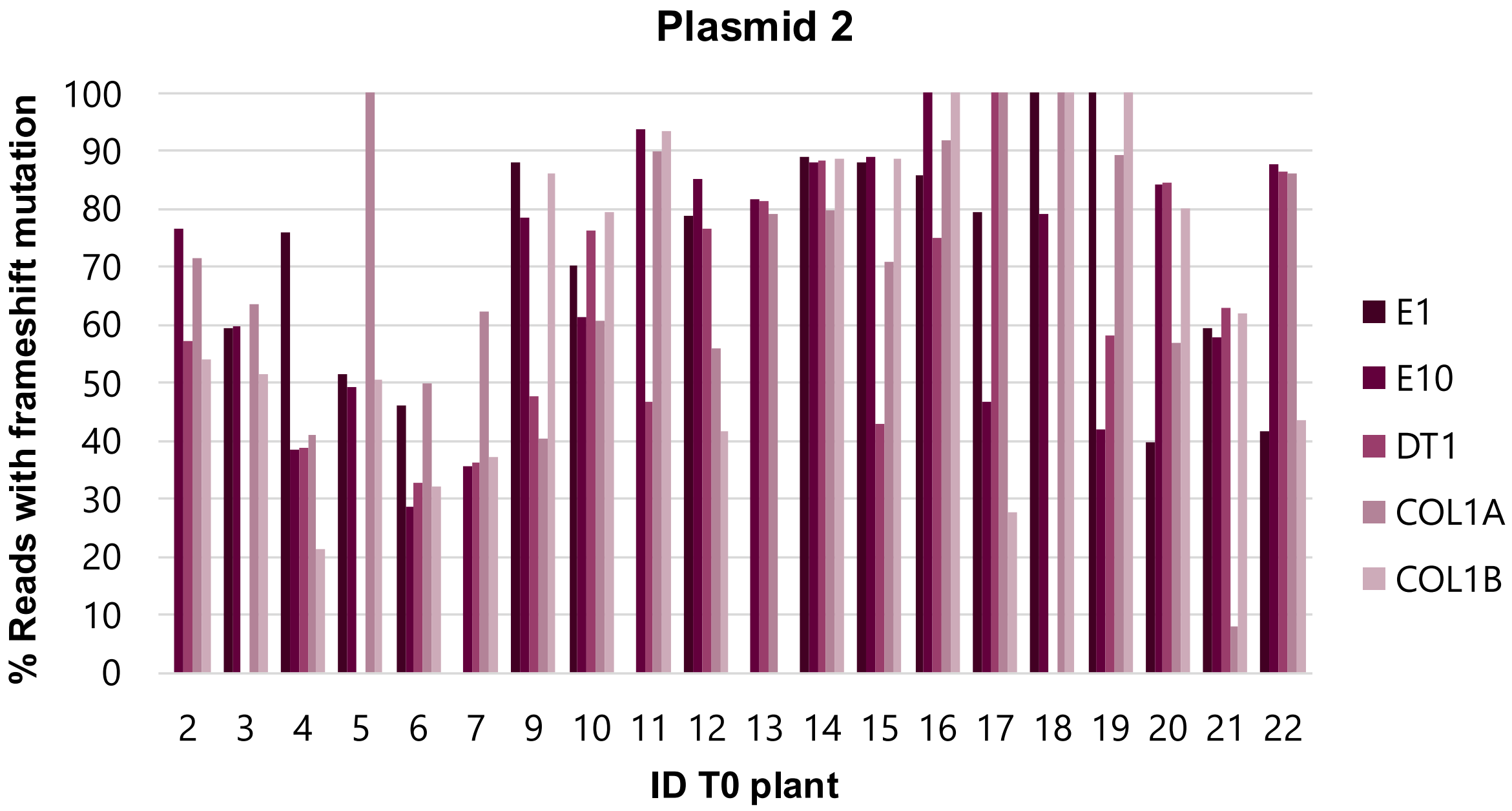


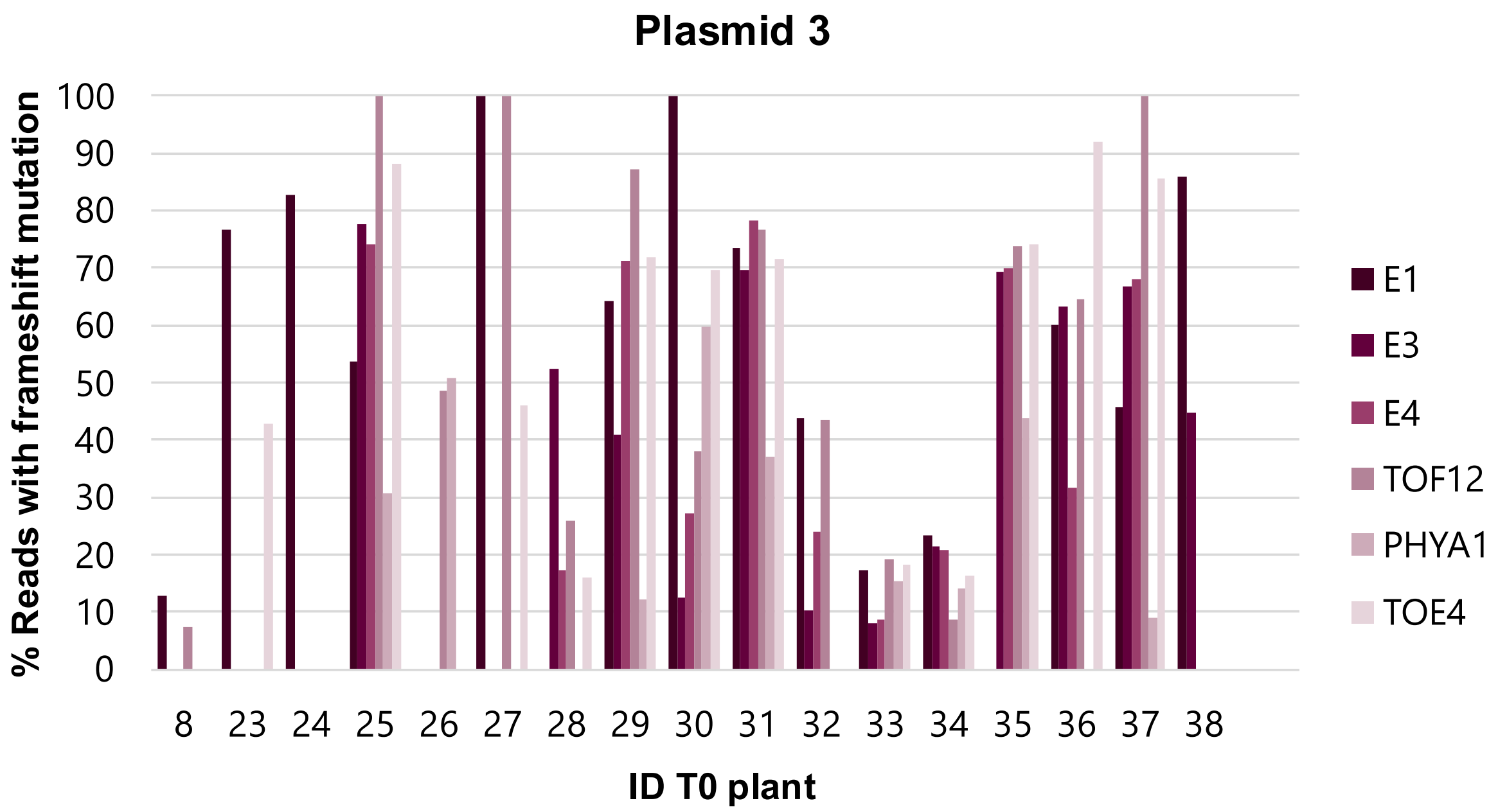


**Figure S5. Illumina sequencing summary for T0 plants transformed with Plasmid 2 and Plasmid 3.** The chart illustrates the percentage of reads displaying frameshift mutations or indels exceeding 27 base pairs (bp) for each targeted gene. T0 plants 2 to 8 are 91B42 genotype and 9 to 38 are 93Y21 genotype.

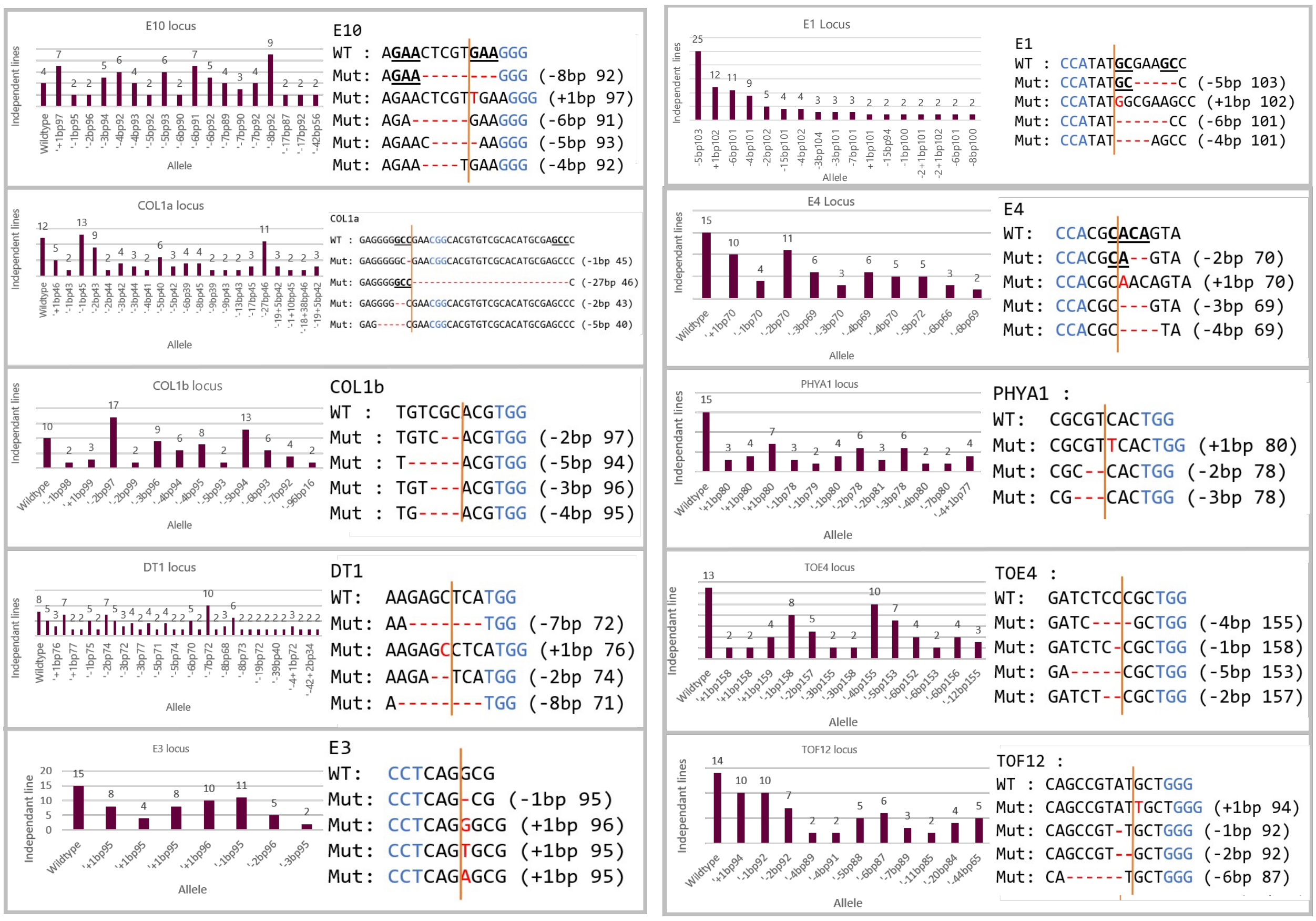


**Figure S6. Mutations obtained at targeted loci.**
Left panel: Number of independent T1 lines where the mutation was found at least twice. Right panel: Sequence of the mutations obtained the most frequently. PAM in blue, cutting site in orange, mutation in red. WT: Wild-type sequence, Mut: mutant sequence, bp: base pair.

A

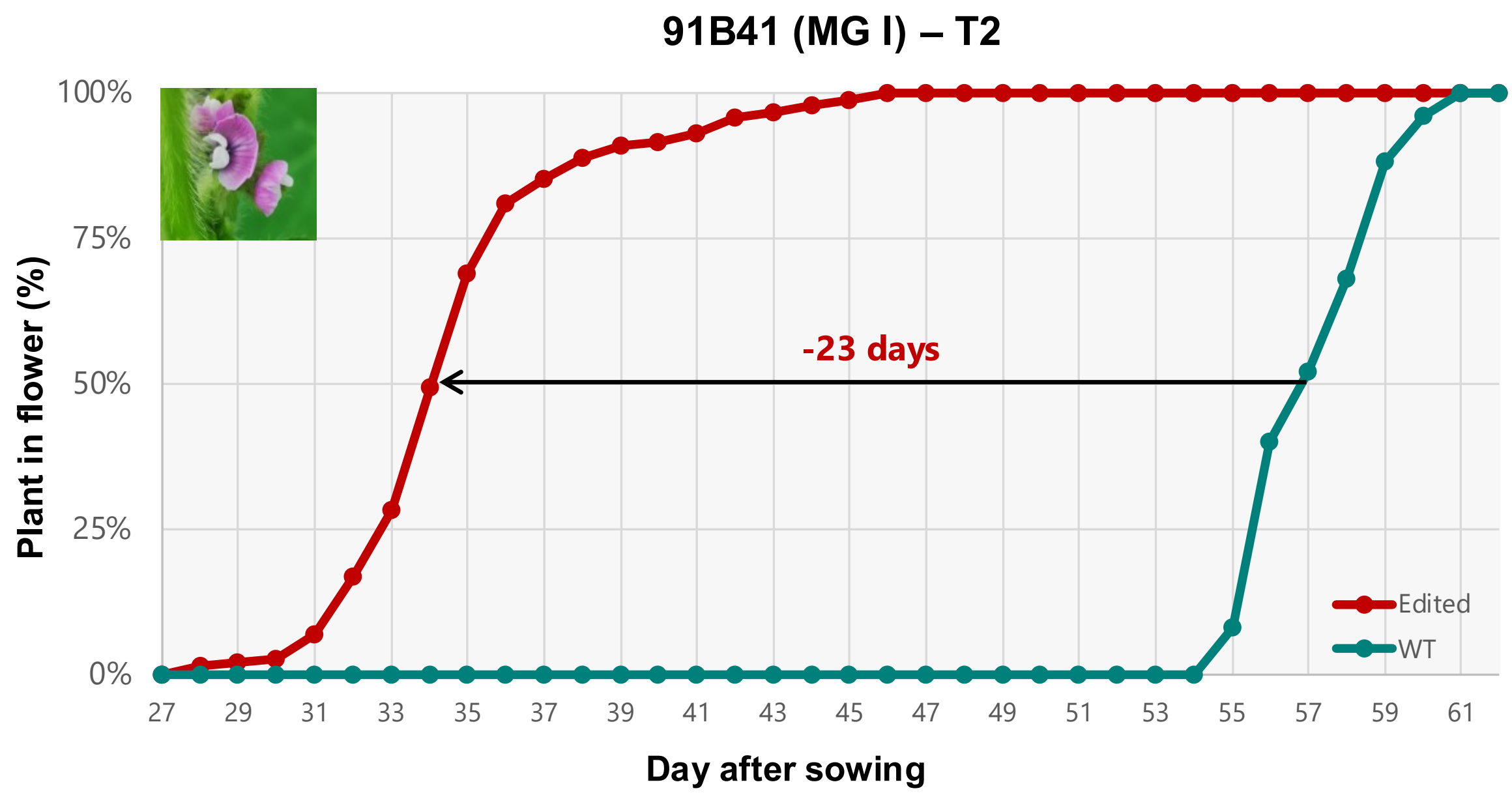


B

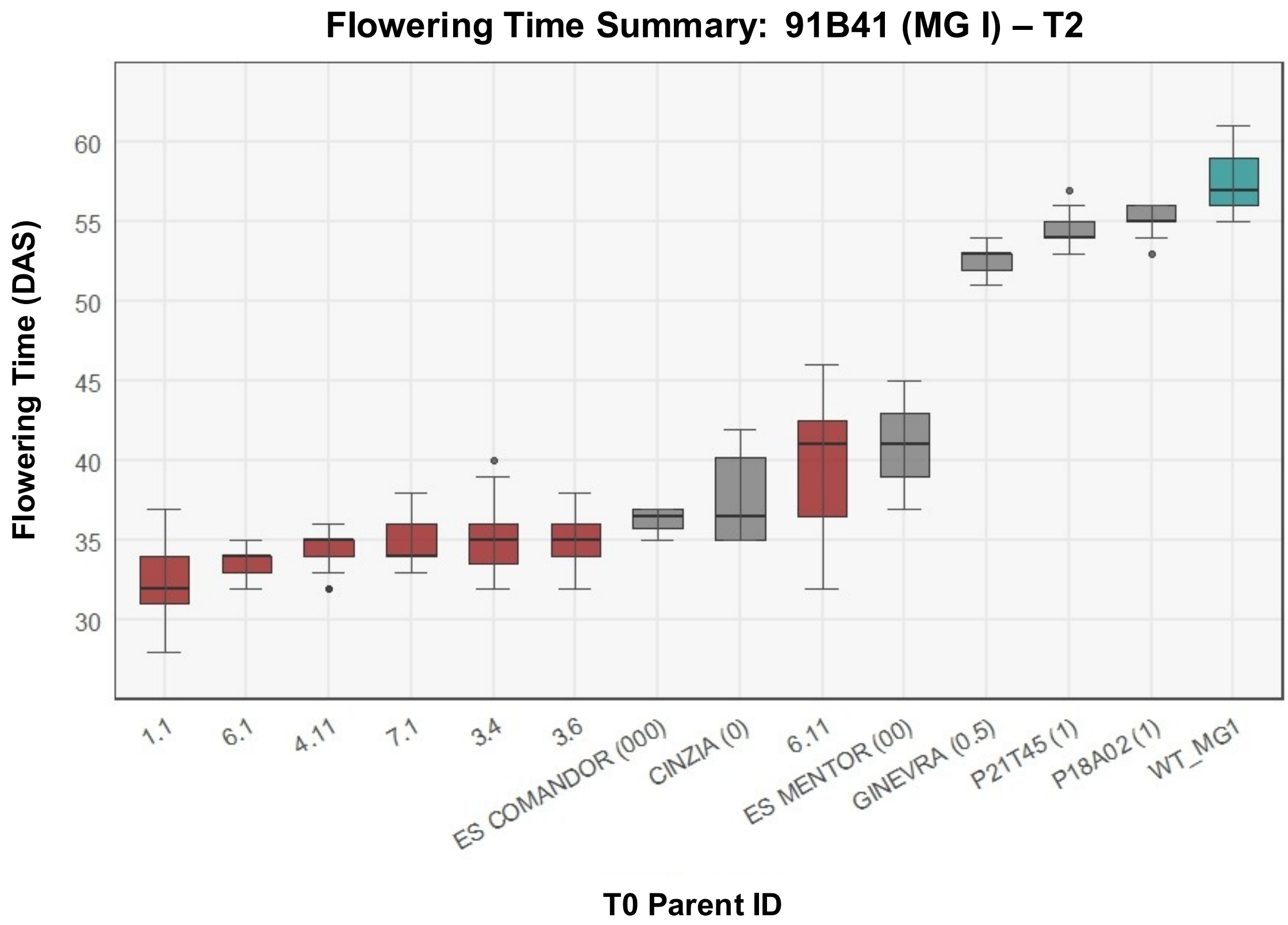


**Figure S7. T2 flowering time analysis for 91B42 population.**
**(A)** Percentage of T2 plants flowering as a function of the number of days after sowing for the 91B42 (MG I) WT population (n=25) and the edited T2 progeny population (n=119). **(B)** Flowering time obtained for each T2 line originating from for 91B42 genotype. Each boxplot corresponds to the T2 progeny of one T1 plant. Edited lines are in red, wild type in blue, and external commercial control in grey. The numbers in bracket correspond to the maturity group of the commercial controls. DAS: Days after sowing.

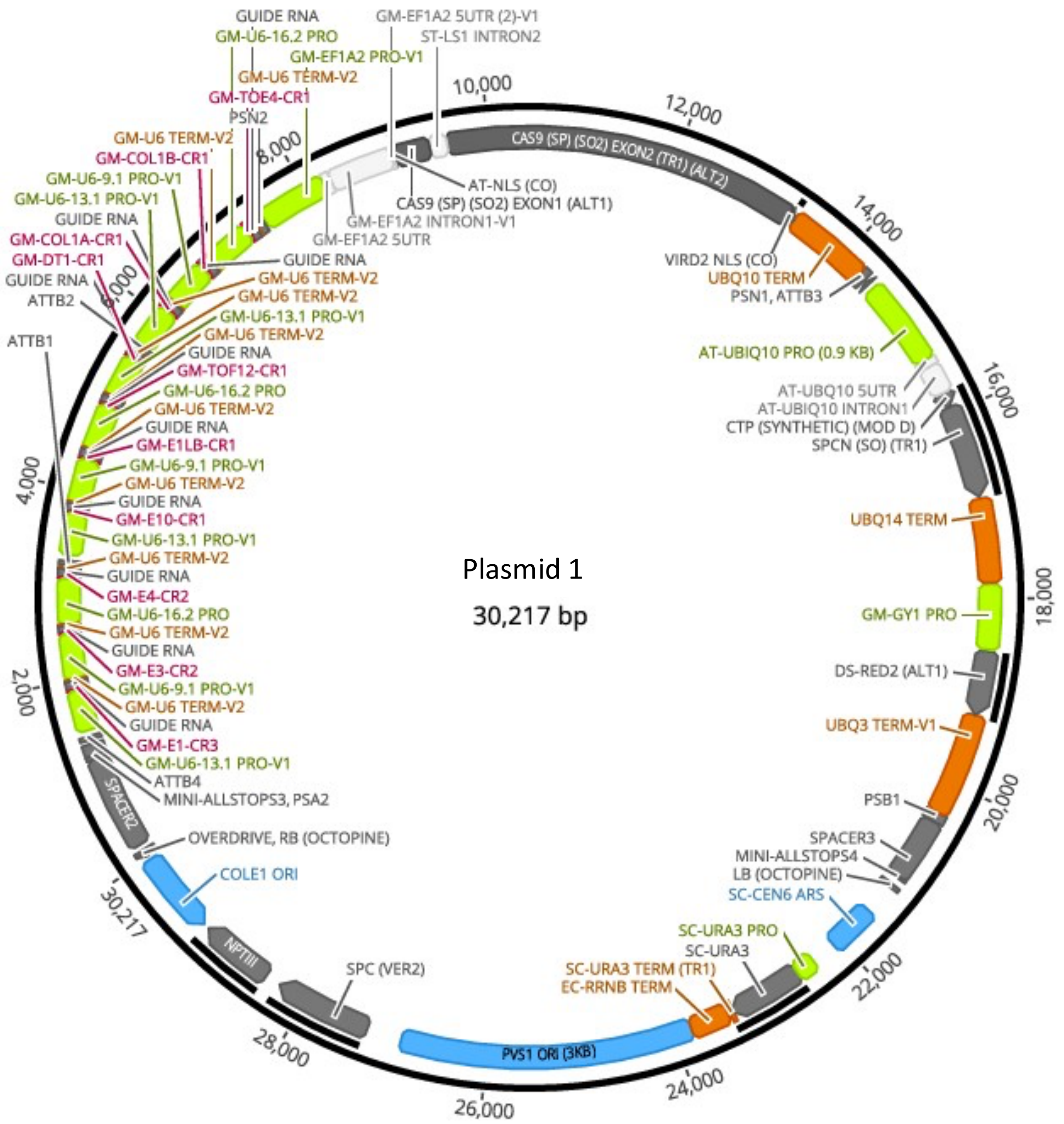


**Figure S8. Map of Plasmid 1.**

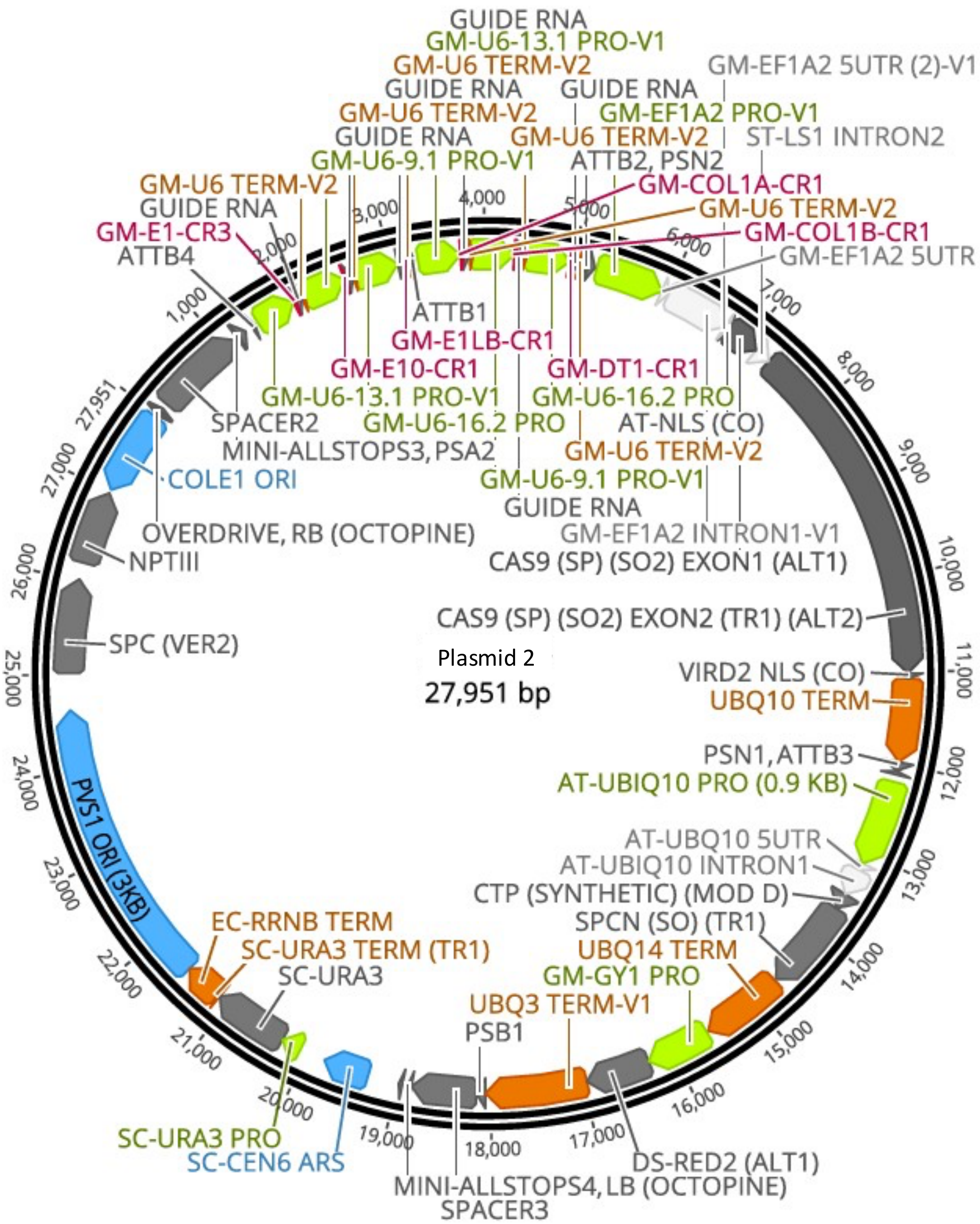


**Figure S9. Map of Plasmid 2.**

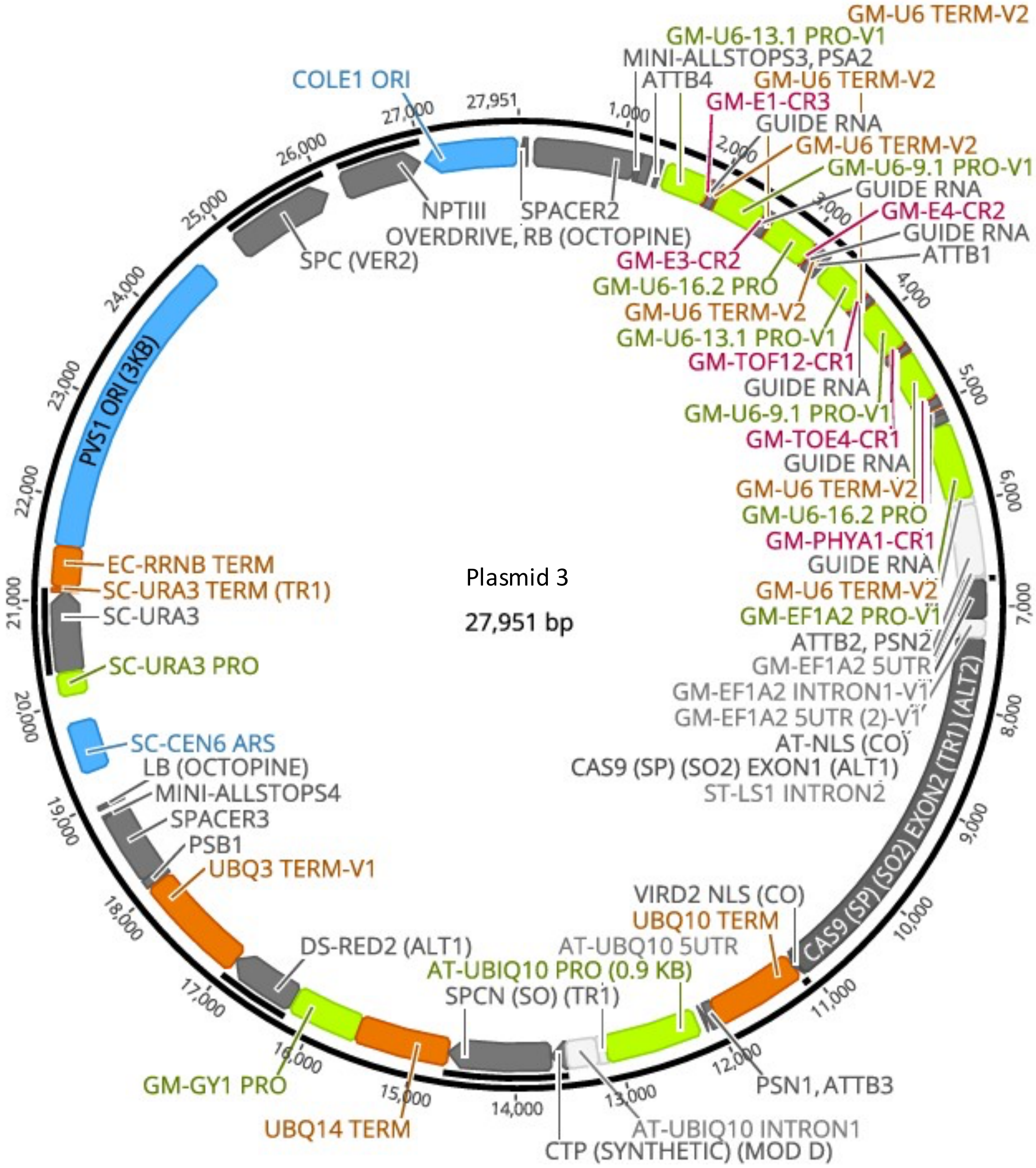


**Figure S10. Map of Plasmid 3.**

(A) (B) (C)

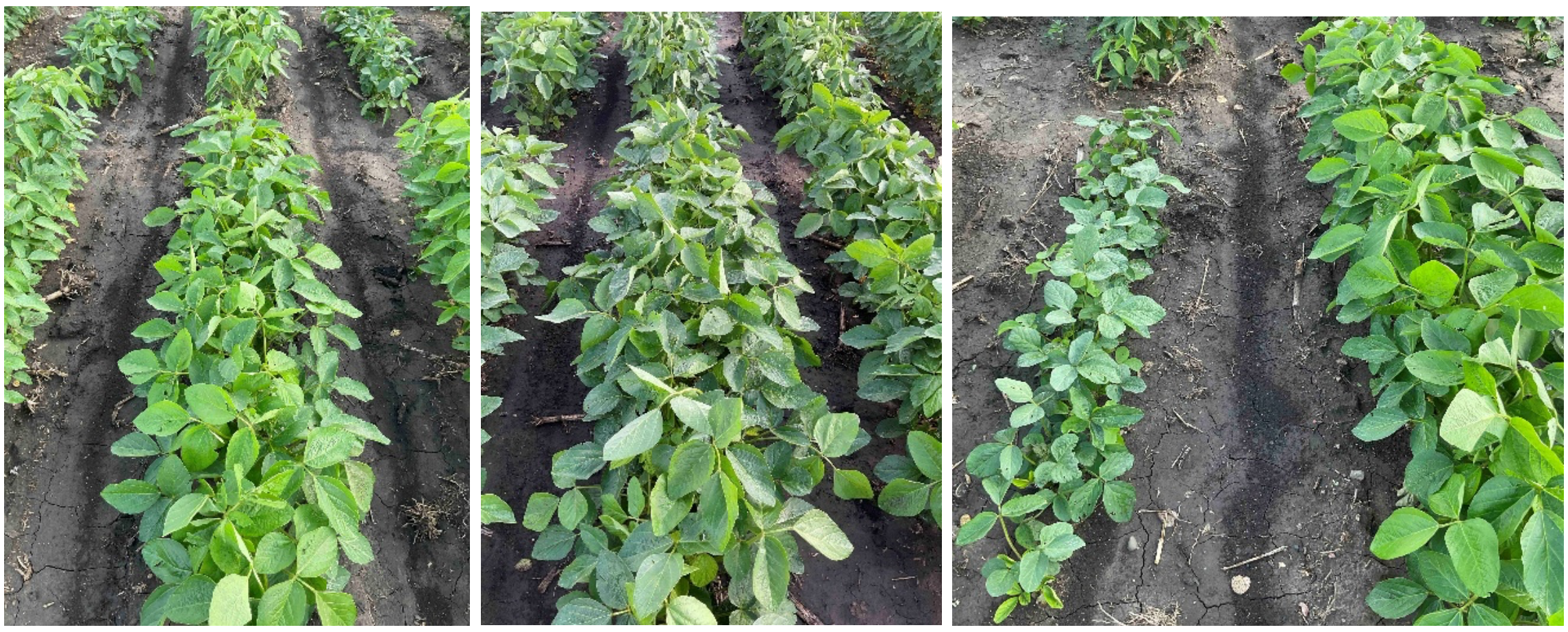

**Figure S11. Representative picture of soybean in the field**. (A) 91B42 WT. (B) Edited line (line #39) (C) DT1 knock out on the left (line #30), 91B42 WT on the right.
