# Supplementary material for: Targeted allelic diversification of flowering genes via CRISPR-Cas enables the development of multiple early-flowering soybean lines
Source: Theor Appl Genet. 2026 Sep 29;139(10):286. doi: 10.1007/s00122-026-05400-9 (PMC13623812; doi:10.1007/s00122-026-05400-9)
Supplement: Supplementary file 2 [file 122_2026_5400_MOESM5_ESM.pdf]

Table S1. Flowering repressor genes selected as target to obtain early flowering soybean lines.

Locus	ID[a]
**E1**	Glyma.06G207800
**E3**	Glyma.19G224200
**E4**	Glyma.20G090000
**E10 (GmFT4)**	Glyma.08G363100
**E1La**	Glyma.04G156400
**E1Lb**	Glyma.04G143300
**Dt1**	Glyma.19G194300
**GmCOL1a**	Glyma.08G255200
**GmCOL1b**	Glyma.18G278100
**TOE4a**	Glyma.15G044400
**GmPHYA1**	Glyma.10G141400
**Tof12 (Gp12, q-FT12-1)**	Glyma.12G073900

[a] Based on Phytozome G.max Wm82.a6.v1 sequence

Table S2: Alleles combination of each genotype of 91B42, 93Y21, and reference cultivar William 82 at the targeted loci.

Cultivar	Genes											
	E1	E3	E4	E10	E1La	E1Lb	Tof12	Dt1	GMCOL1a	GMCOL1b	TOE4a	GMPHYA1
**W82**	e1-as	E3-Ha	E4	E10	E1La	E1Lb	tof12-1	Dt1	GmCOL1a	GmCOL1b	TOE4a	GmPHYA1
**91B42**	e1-as	E3-Ha	E4	E10	E1La	E1Lb	tof12-1	Dt1[b]	GmCOL1a	GmCOL1b[c]	TOE4a	GmPHYA[b]
**93Y21**	e1-as	E3-Ha	E4	E10	na[a]	na[a]	tof12-1	Dt1	GmCOL1a	GmCOL1b[c]	TOE4a	GmPHYA1

[a] na: not analyzed as E1La and E1Lb are not targeted in 93Y21

[b] synonymous SNPs compare to William 82 without consequence on the peptide sequences.

[c] synonymous SNPs compare to William 82 and one non synonymous leading to an amino acid change (D216E).

Table S3: CRISPR target sequences and number of potentials off-target sites.

Gene	Protospacer + PAM (CRISPR Target) [a]	Spacer (gRNA)	Off-target sites 91B42[b]	Off-target sites 93Y21[b]
E1	**A**AAGTTAGAGGCTTCGCATA TGG	GAAGTTAGAGGCTTCGCATA	0/0/0/2/51	0/0/0/2
E3	**A**ACAAAAAGCGTGTCGCCTG AGG	GACAAAAAGCGTGTCGCCTG	0/0/0/1/23	0/0/0/1
E4	**T**GTTCGCCATGTACTGTGCG TGG	GGTTCGCCATGTACTGTGCG	0/0/0/1/5	0/0/0/1
E10	**A**GAGAAACAGAACTCGTGAA GGG	GGAGAAACAGAACTCGTGAA	0/0/0/12/186	0/0/0/12
E1La<br>E1Lb	**A**ATCCTAAGTAGACTCTCGC TGG	GATCCTAAGTAGACTCTCGC	0/0/1/3/38	NA
Tof12	**T**CAAGAGCCAGCCGTATGCT GGG	GCAAGAGCCAGCCGTATGCT	0/0/0/1/16	0/0/0/1
Dt1	**C**GGTGGAAGGGAAGAGCTCA TGG[c]	GGGTGGAAGGGAAGAGCTCA	0/0/0/7/131	0/0/2/12
COL1a	**A**CACGGAGGAGGGGGCCGAA CGG	GCACGGAGGAGGGGGCCGAA	0/0/0/3/19	0/0/0/3
COL1b	GTGGAGGCGAGTGTCGCACG TGG	GTGGAGGCGAGTGTCGCACG	0/0/2/2/48	0/0/2/2
TOE4a	GAATTTAATGGATCTCCCGC TGG	GAATTTAATGGATCTCCCGC	0/0/0/3/33	0/0/0/3
PHYA1	GCAATTGTCCATCGCGTCAC TGG	GCAATTGTCCATCGCGTCAC	0/0/0/3/17	0/0/0/2

[a] Bases in bold font in the protospacer are replaced with G in the gRNA spacer to ensure proper transcription initiation following the GmU6 promoter.

[b] Number of potential off-target sites were quantified by counting the number of loci with 0, 1, 2, and 3 mismatches compared to the target

[c] In 93Y21, the G in second position is replaced by an A.

Table S4. Number of explants and corresponding T0 and T1 seed set of Agrobacterium-mediated transformation with Plasmid 1, 2, and 3.

Plasmid	Genotype	Number of explants	T0 send to greenhouse	T0 which gave seeds	T1 seeds obtained
**1**	91B42	1000	17	1	1
**2**	91B42	2000	40	6	51
	93Y21	500	20	14	239
**3**	91B42	2000	38	1	3
	93Y21	500	20	16	434

Table S5. Analyses of 93Y21 (MGIII) flowering time compared to wild-type population on T1 generation.

Line	N	Mean flowering time (days after sowing)	Flowering acceleration compared to WT (days)	Significative[a]
**37**	3	37	-24.5	**
**14**	5	37.2	-24.3	****
**29**	2	37.5	-24	ns
**35**	34	37.6	-23.9	****
**19**	22	38.8	-22.7	****
**18**	1	39	-22.5	NA[b]
**22**	1	39	-22.5	NA[b]
**15**	5	39.2	-22.3	**
**23**	2	39.5	-22	ns
**27**	9	39.7	-21.8	****
**9**	2	40	-21.5	ns
**30**	8	41.1	-20.4	***
**12**	5	41.2	-20.3	**
**24**	18	42.1	-19.4	****
**16**	15	42.5	-19	****
**20**	17	42.7	-18.8	****
**25**	8	42.8	-18.8	**
**10**	12	44.2	-17.3	****
**31**	6	44.3	-17.2	ns
**17**	18	44.4	-17.1	****
**28**	16	47.1	-14.4	***
**11**	25	50.1	-11.4	***
**13**	8	50.1	-11.4	ns
**21**	7	57.3	-4.2	ns
**26**	44	59.4	-2.1	ns
**34**	53	59.8	-1.7	ns
**33**	29	61.3	-0.2	ns
**W82**	11	63.4	1.9	ns
**WT_93Y21**	44	61.5	0	/

[a] Comparison of flowering time between edited groups and WT groups. (*) Dunn's test p-v<0.05; (**) Dunn's test p-v<0.01; (***) Dunn's test p-v<0.001; (****) Dunn's test p-v<0.000, (ns) Dunn's test not significant.

[b] NA: non-applicable due to low N

Table S6. Analyses of genotype 91B42 (MG I) flowering time compared to wild-type population for T1 generation.

Line	N	Mean flowering time (days after sowing)	Flowering acceleration compared to WT (days)	Significant[a]
**1**	1	36	-16.1	NA[b]
**2**	2	44	-8.1	ns
**3**	6	38	-14.1	**
**4**	10	38.9	-13.2	**
**5**	1	39	-13.1	NA[b]
**6**	10	37.4	-14.7	****
**7**	1	36	-16.1	NA[b]
**8**	1	54	1.9	NA[b]
**WT_91B42**	21	52.1	0	/

[a] Comparison of flowering time between edited groups and WT groups. (*) Dunn's test p-v<0.05; (**) Dunn's test p-v<0.01; (***) Dunn's test p-v<0.001; (****) Dunn's test p-v<0.000, (ns) Dunn's test not significant.

[b] NA: non-applicable due to low N.

Table S7. Overview of the type of mutations obtained for each target locus.

Loci	Number of edited Alleles created	Number of edited alleles causing frameshift mutation	Number of edited alleles causing in-frame mutation	Potential C-NHEJ	Potential Alt-EJ	Unknown
**E1**	46	28	18	6	32	8
**COL1a**	53	35	18	3	29	21
**COL1b**	31	18	13	3	28	0
**E10**	37	31	6	2	32	3
**Dt1**	73	48	25	7	47	19
**E3**	23	16	7	5	13	5
**E4**	39	24	15	2	31	6
**PHYA1**	28	22	6	7	14	7
**TOE4a**	36	21	15	4	27	5
**Tof12**	23	17	6	1	21	1
**TOTAL**	389	260	129	40	274	75
**%**		67%	33%	10%	70%	19%

Table S8. Analyses of genotype 93Y21 (MG III) flowering time compared to wild-type population on T2 generation.

Line	N	Mean flowering time (days after sowing)	Flowering acceleration compared to WT (days)	Significant[a]
**10.14**	33	35.8	-26.1	****
**11.19**	4	54.5	-7.4	ns
**11.53**	3	54.3	-7.6	ns
**11.6**	18	53.3	-8.7	ns
**12.2**	11	37.8	-24.1	****
**13.1**	20	52.7	-9.2	ns
**13.11**	29	52.3	-9.6	ns
**13.4**	33	53.5	-8.5	ns
**13.6**	37	53.2	-8.8	ns
**13.7**	21	54.5	-7.5	ns
**14.3**	47	36.3	-25.6	****
**14.5**	3	36.7	-25.3	*
**15.6**	37	35.2	-26.8	****
**16.14**	7	37.9	-24.1	***
**16.15**	12	36	-25.9	****
**16.4**	18	49.4	-12.5	ns
**19.1**	21	40.7	-21.2	****
**19.10**	15	38.3	-23.7	****
**19.11**	17	42	-19.9	**
**19.12**	8	35.1	-26.8	****
**20.1**	9	49.8	-12.2	ns
**20.18**	31	35.2	-26.7	****
**20.3**	16	36	-25.9	****
**20.9**	11	45.1	-16.9	ns
**21.12**	9	49.1	-12.8	ns
**21.2**	3	38.7	-23.3	ns
**21.4**	61	38.8	-23.1	****
**23.3**	12	43.2	-18.8	ns
**24.19**	32	42.4	-19.5	***
**24.3**	18	42.1	-19.9	**
**30.17**	7	43.6	-18.4	ns
**31.15**	18	42	-19.9	**
**34.10**	22	481	-13.8	ns
**35.3**	22	39.5	-22.5	****
**35.32**	9	41.6	-20.4	*
**35.39**	18	36.6	-25.4	****
**35.5**	11	41.8	-20.1	*
**37.1**	6	40.2	-21.8	ns
**38.2**	14	40.7	-21.2	***
**CINZIA (0)**	6	37.7	-24.3	***
**ES COMANDOR (000)**	4	36.3	-25.7	***
**ES MENTOR (00)**	7	41	-20.9	*
**GINEVRA (0.5)**	22	52.8	-9.1	ns
**W82**	10	62,4	0,5	ns
**WT_93Y21**	18	61,9	0,0	

[a] Comparison of flowering time between edited groups and WT groups. (*) Dunn's test p-v<0.05; (**) Dunn's test p-v<0.01; (***) Dunn's test p-v<0.001; (****) Dunn's test p-v<0.000, (ns) Dunn's test not significant.

Table S9. Analyses of genotype 91B42 (MG I) flowering time compared to wild-type population for T2 generation.

Line	N	Mean flowering time (days after sowing)	Flowering acceleration compared to WT (days)	Significant[a]
**1.1**	23	32,2	-25,3	****
**6.1**	13	33,4	-24,1	****
**4.11**	37	34,5	-23	****
**7.1**	13	34,8	-22,7	****
**3.4**	19	35,1	-22,4	****
**3.6**	14	35,1	-22,4	****
**ES COMANDOR (000)**	4	36,3	-21,2	ns
**CINZIA (0)**	6	37,7	-19,8	ns
**6.11**	23	39,9	-17,6	**
**ES MENTOR (00)**	7	41	-16,5	ns
**GINEVRA (0.5)**	22	52,8	-4,7	ns
**P21T45 (1)**	13	54,5	-3	ns
**P18A02 (1)**	13	55,1	-2,4	ns
**WT_91B42**	25	57,5	0	/

[a] Comparison of flowering time between edited groups and WT groups. (*) Dunn's test p-v<0.05; (**) Dunn's test p-v<0.01; (***) Dunn's test p-v<0.001; (****) Dunn's test p-v<0.000, (ns) Dunn's test not significant.

Table S10. PCR primers used for genotyping of the target genes.

Loci	Primer Forward	Primer Reverse
**E1**	CAAAGCCCATCAAAGTTCACG	TGAAACTCCCTTATTGTTCATCTCC
**E1La**	TGAACAAGGGAGTTTCAACAAC	CCTTCTTCAGTTTCTGCAGCTC
**E1lb**	TGAACAAGGGAGTTTCAACAAC	CCTTCTTCAGTTTCTGCAGCTC
**E3**	TGAGGATGATCATGGGGAAG	AAGCACATTCACATGCTTTGC
**E4**	GATGAAAAACTCCCATTTGATTTGAC	CCAATCCCCAAAGTCTCTTCC
**E10**	TCTTGGTTTGTTGACGATTTGG	ATGGACCCCCTTGTTCTTGG
**PHYA1**	CGGACGTTTCGCTTCTTAACC	CATGTTCCCACTAGGCAATGATTGC
**TOE4a**	GACGTGTTCGCCTTCAGTTTC	GACCTCTCCTACTCTTCTTCACCTG
**Tof12**	TGGTTGAAGATGGAAACAATGG	TCCACCAGCAAAACTTTTATCG
**COL1a**	CAGAGGAAGGCGGTGTGG	CGAATGTTGGATGGAGAAGC
**COL1b**	GTCGGCCTTGCAGAGGAAC	CAGAACACTAACTCCCCCAATG
**Dt1**	AGTGTAAAGAAGGACCTCATATCACC	ACCACAAGCACAAAAATGACTG

Table S11. PCR mix and conditions for genotyping of the targeted genes.

PCR for Illumina sequencing

PCR 1 Reagents	Volume (µl)
DNA (10ng/µl)	5
Forward/Reverse Primer mix with linker sequence (10µM)	2
2X Phusion Flash Master Mix	10
Nuclease Free Water	3

PCR 1 steps	Time (s)	Temperature (°C)	
1	30	98	
2	5	98	X 29
3	10	63.8	
4	15	72	
5	60	72	
6	-	4	

PCR 2* Reagents	Volume (µl)
Eluted DNA from PCR 1	5
Amplicon Indexes (10µM)	2
2X Phusion Flash Master Mix	10
Nuclease Free Water	3

PCR 2 steps	Time (s)	Temperature (°C)	
1	10	98	
2	1	98	X 20
3	5	63.8	
4	60	72	
5	60	72	
6	-	4	

PCR for Sanger sequencing

Reagents	Volume (µl)
DNA	1
10x PCR Buffer	2
MgCl2	2
dNTP	0.2
Forward/Reverse Primer mix (10µM)	2
Taq polymerase (rec)	0.2
Nuclease Free Water	12.6

PCR steps	Time (s)	Temperature (°C)	
1	300	94	
2	20	94	X 35
3	30	58	
4	30	72	
5	300	72	
6	-	10	

*PCR 2 add specific adaptors (or indexes) to the amplicons to allow pooling of all samples
